# The Sound of Voice: Voice-Based Categorization of Speakers’ Sexual Orientation within and across Languages

**DOI:** 10.1371/journal.pone.0128882

**Published:** 2015-07-01

**Authors:** Simone Sulpizio, Fabio Fasoli, Anne Maass, Maria Paola Paladino, Francesco Vespignani, Friederike Eyssel, Dominik Bentler

**Affiliations:** 1 Department of Psychology and Cognitive Science, University of Trento, Trento, Italy; 2 Fondazione Marica De Vincenzi ONLUS, Rovereto (TN), Italy; 3 Department of Developmental Psychology and Socialization, University of Padua, Padua, Italy; 4 Center of Excellence–Cognitive Interaction Technology, University of Bielefeld, Bielefeld, Germany; 5 Psychology Faculty, New York University Abu Dhabi, Abu Dhabi, United Arab Emirates; Northwestern University, UNITED STATES

## Abstract

Empirical research had initially shown that English listeners are able to identify the speakers' sexual orientation based on voice cues alone. However, the accuracy of this voice-based categorization, as well as its generalizability to other languages (language-dependency) and to non-native speakers (language-specificity), has been questioned recently. Consequently, we address these open issues in 5 experiments: First, we tested whether Italian and German listeners are able to correctly identify sexual orientation of same-language male speakers. Then, participants of both nationalities listened to voice samples and rated the sexual orientation of both Italian and German male speakers. We found that listeners were unable to identify the speakers' sexual orientation correctly. However, speakers were consistently categorized as either heterosexual or gay on the basis of how they sounded. Moreover, a similar pattern of results emerged when listeners judged the sexual orientation of speakers of their own and of the foreign language. Overall, this research suggests that voice-based categorization of sexual orientation reflects the listeners' expectations of how gay voices sound rather than being an accurate detector of the speakers' actual sexual identity. Results are discussed with regard to accuracy, acoustic features of voices, language dependency and language specificity.

## Introduction

Sometimes when you want to believe so badly,

you end up… looking too hard.

(*X-Files*, *Season 2 –Episode 5*)

Overhearing a voice often leads individuals to spontaneously categorize the speaker as a member of a specific social group. A few seconds of listening are enough to form impressions about a person’s gender, ethnicity, age, and even about his/her personality [1]. In the case of sexual orientation (from now on SO), it has often been argued that people possess an ability to recognize a man’s SO on the basis of subtle indirect cues such as walking style or facial features [2]. Recent studies have suggested that this “detection skill” may function only on the basis of acoustic cues, meaning that people infer the SO of male speakers from voice alone [3, 4, 2]. Interestingly, categorization of SO seems to be more accurate when based on vocal than facial features [5]. However, acoustic cues *per se* can be misleading [6, 7] since they are affected by both anatomical aspects (speaker's size, shape, and physical conditions; [8]) and social expectations (e.g., social group membership or gender role; [9]). The reliance on cultural rather than anatomical cues has been shown in other lines of research, such as when adults identify the sex of children based more on behavioral than on the basis of actual anatomical differences [10, 11]. Moreover, the absence of any physiological correlates of SO makes its voice-based categorization even more problematic. Whereas male and female voices differ from each other as a consequence of physical (e.g., height) and biological features (e.g., testosterone; [12, 13]), it is less clear on which physiological grounds gay and heterosexual male voices should be distinguishable. Among others, Zimman [14] has recently remarked that rather than using actual cues, perceivers may draw inferences about SO from the degree to which an individual’s speech style deviates from typical heterosexual voices. Thus, rather than reflecting actual differences in speech style, such voice-based categorization seems to be driven by beliefs about what gay vs. heterosexual voices sound like.

The present research aims to address the issue of accuracy of the voice-based categorization of SO and to contribute to this line of work by investigating this process across two different languages not investigated in prior research.

### Voice-based categorization of speakers’ sexual orientation

Since the seminal study by Gaudio [3], the issue of voice-based categorization of SO has stirred much discussion and produced heterogeneous findings. Initial research provided evidence for the fact that listeners are accurate in judging the speakers' SO on the basis of their voice [3, 5]. However, Smyth and colleagues [6] have suggested that listeners’ judgments are often inaccurate. In fact, correct recognition seems generally driven by a small subset of voices that are consistently judged as gay- or heterosexual-sounding. In line with this observation, recent studies on this voice-based categorization process have moved attention toward *perceived* speakers’ SO, analyzing how listeners categorize a speaker as gay or heterosexual regardless of the accuracy of these judgments [15, 4, 6]. Listeners may have expectations of how gay versus heterosexual males speak and, hence, categorize voices according to whether they do or do not match such expectations (on this issue, see [15]).

Studies on voice-based categorization of SO have mainly examined which acoustic cues (e.g., vowel duration) drive the listener’s categorization. However, only few studies have also investigated whether there is any actual acoustic difference in the speech of gay- and heterosexual-speakers. On this issue, Pierrehumbert and colleagues [16] analyzed the speech of English speakers and reported differences in the first-and second-formant frequency of vowels /ɑ/, /i/, and /u/. Similarly, Munson and colleagues [4] reported that English self-identified gay- and heterosexual-speakers differed in the way they produced the first-formant frequency of /ae/ and /ε/, and the spectral skewness of /s/.

Turning to the relation between acoustic signal and listener’s categorization of speakers' SO, studies addressing this issue have reported mixed results. On the one hand, both Gaudio [3] and Smyth and colleagues [6] investigated the relation between voice pitch and listeners' perception of speakers' SO, but both studies failed to find any significant correlation between the two. On the other hand, Munson and colleagues [4] investigated several acoustic cues related to the speech signal and reported that listeners' perception was driven by first-formant frequency of front vowels and the spectral skewness of /s/ (for the similar results, see also [15]). Particularly telling is the work of Munson and colleagues that allows one to directly compare the acoustic cues as a function of actual and perceived SO. The comparison shows that there is overlap between the two types of information as both self-identified gay- and heterosexual-speakers, and sounding gay- and heterosexual-speakers differed in terms of first-formant frequency of frontal vowels and spectral skewness of /s/.

To summarize, research has shown that there is a relation between the way speakers’ speak (acoustic features) and how they are categorized by listeners. Although interesting and informative with regard to the process of voice-based categorization, these studies have provided mixed results regarding the accuracy of this categorization process. Indeed, previous research has mainly focused on the acoustic cues linked to either actual or perceived SO in speech (except for [4]). This raises the question of which cues are actually used by listeners to make inferences and whether these cues are the same that objectively distinguish gay and heterosexual voices.

### Language-specificity and language dependency

The Achilles' heel of research on categorization of SO on the basis of voice is the restricted linguistic context in which studies have been conducted. Previous research has involved almost exclusively English speakers and listeners. As a consequence, it remains unclear whether the voice-based categorization of SO is *language-dependent*–that is, whether it occurs in English, but not in other languages. Moreover, to our knowledge, it is also not clear whether this process is *language-specific*–that is, whether listeners recognize the SO only for individuals who speak the same language or also for those speaking a foreign language. Both issues are crucial for our understanding of this voice-based categorization process as either a language-specific process or a universal phenomenon.

According to Munson and Babel [17] the question of *language-dependency* is “absolutely essential” (p. 436) for a number of reasons. Most importantly, the vocal expression of SO is necessarily constrained by the parameters of any given language (e.g., type and number of vowels and flexibility of their use), suggesting that (a) the expression of SO may be easier to emerge in some languages than in others and (b) that different acoustic cues may be used in different languages to express (and interpret) SO. In line with this idea Zimman [14] has suggested that even differences in dialects among English speakers may produce distinct perceptions of voices as gay-sounding (see also, [18, 19]). By extension, one may suspect that languages, even more so than dialects, may influence how listeners categorize speakers. Hence, being exposed to languages that possess a higher frequency of “gay-related” acoustic cues should increase the likelihood, and possibly the accuracy, of distinguishing between gay and heterosexual speakers. Moreover, the construal of gender and SO varies greatly across cultures [19], as does the stigma associated with gay membership, which in turn is likely to affect those gender-related speech patterns that are under speaker’s control. The investigation of different languages therefore becomes essential to understand whether voice based categorization of SO is a *language-dependent* process, being proper for the English language (and the North-American context) only, or whether it is generalizable across languages. If similar patterns of voice-based categorization and of related acoustic cues were to emerge from the analysis of different languages, this would suggest that this process is a more general, possibly even universal, phenomenon. In contrast, a cross-linguistic difference would suggest that this process is a by-product of a specific cultural and language environment. To our knowledge, only one study [5] has investigated the ability to detect SO in a language other than English, namely Czech. According to the authors, results suggested an overall good accuracy of judgments about targets’ SO among a sample of heterosexual female and gay male listeners [5].

Turning to the question of *language-specificity*, Schwieter [20] has recently stressed the need to investigate the ability of listeners to detect the SO of speakers of different languages in order to pinpoint how this voice-based categorization operates. To date, only one study has addressed the issue of cross-cultural categorization of SO. In this study, Valentova and colleagues [21] have examined how American and Czech participants categorized SO of people of their own and of the other country. Participants watched short videotapes and indicated the targets' SO based on acoustic, visual, and gestural features. The authors found an above-chance relation between raters' judgment and self-defined targets' SO, although this relation was stronger when targets and raters shared the same nationality. The study provides first evidence of cross-cultural categorization of gay men, but the findings still remain inconclusive, given that raters simultaneously made use of both acoustic and visual cues. On one side, it is possible that the availability of visual cues may have increased accuracy above and beyond the inferences drawn from acoustic cues alone. Indeed, several studies have shown that visual cues such as face and gesture are particularly informative about SO [22, 23]. On the other side, it is possible that raters might have derived their judgments mainly from vocal information. This argument is in line with a recent study showing that voice, but not face, was a meaningful cue in categorization of SO [5]. Thus, it remains currently unclear whether people categorize others as gay vs. heterosexual on the basis of voice alone when confronted with foreign language speakers. If we want to understand the generality of voice-based categorization of SO, both language-dependence and language-specificity need to be investigated systematically. The present study provides a first step in this direction.

### Aims of the present research

Starting from the ongoing debate outlined above, the current study addresses four main questions: First, we investigated whether voice-based categorization is, to some degree, accurate or whether it is purely driven by perceivers' expectancy. Thus, we tested whether heterosexual listeners are able to correctly identify male speakers' SO from voice alone or whether they base their judgments on what the speakers sound like, regardless of the actual SO of the speaker (in which case categorization would be expectancy-driven). To address this issue, we ran five experiments in which we asked participants to listen to male voices and to categorize the speakers' SO using an explicit or an indirect measure.

Second, to investigate the question of *language-dependency*, we considered speakers and listeners of two different languages, namely German and Italian. This allowed us to also address our third aim, namely *language-specificity* given that our Italian and German participants were asked, in Study 3, to judge the likely SO of both Italian and German speakers.

More importantly for our aims, Italian and German differ at multiple levels. A first difference is that Italian pertains to the Romance languages, whereas German is a West Germanic language, just like English. Note also that both languages differ from Czech (the only language other than English that has been investigated). Czech belongs indeed to a specific West Slavic language group that is very different from other Indo-European languages. Moreover, and most critical to the aims of our research, German and Italian differ with respect to their phonological system and its phonetic realization. German has a larger number of vowels and consonants than Italian [24]; German is also a language with a higher degree of articulatory flexibility than Italian (i.e., it has a stronger tendency to phonological reduction as exemplified by the reduction of unstressed vowels to *schewa*; [24, 25]). Since a relation between SO and vowel cues has been reported in previous research (e.g., [15]), the comparison between German and Italian may help understand to what extent the perception of speakers' SO is affected by specific language features. Thus, although the main goal of this work is not the investigation of acoustic cues of SO, a look at the relation between speakers' self-identification, listeners' judgment and acoustic information will help to shed light on the general issue of voice-based categorization of speakers’ sexual identity.

A fourth aim of the present work is of methodological nature and concerns the different measures used in prior research. Whereas in some studies, listeners were asked to make dichotomous choices regarding the speakers' SO (i.e., heterosexual vs. gay, [6]), other research has used Likert scales that allow respondents to modulate their responses (e.g., [3, 5, 26]). These differences in measurement may in part explain the contradictory findings in the literature, although their influence is difficult to quantify given that studies also differ in other respects (e.g., the voice samples). We therefore varied the measures across studies while keeping other characteristics of the materials, the procedure and the voice samples constant. In Experiment 1A and 1B, participants were asked to make dichotomous choices, combined with a mouse tracking measure that provides an implicit measure of subjective uncertainty. In Experiment 2A and 2B, judgments were expressed on a continuous, Likert-type scale that allowed responses of degree.

To sum up, in the present research, we address four main limitations of previous work on voice-based categorization of SO: First, in all our studies we examine the *accuracy* of this process to understand whether listeners make their judgments on the basis of actual differences between gay versus heterosexual speech productions (reality-driven) or on the basis of presumed, but non-veridical differences (expectancy-driven). This question had received relatively little attention in prior research. Second, we address the issue of *language-dependency* by examining how the voice-based categorization process operates in languages other than English, namely Italian (Experiments 1A and 2A) and German (Experiments 1B and 2B). In doing so, we also examine whether the speakers’ acoustic cues that have been related to listeners’ judgments of SO in English samples, are also present in other languages. Third, for the first time, we address the issue of *language-specificity*. In particular, we report a cross-linguistic experiment (Experiments 3) testing whether the pattern that heterosexual listeners show when categorizing voices of same-language speakers also holds when they categorize voices speaking a foreign language. Fourth, across studies, we employ two different measures to assess listeners’ judgments, while holding the stimulus materials and procedure constant. This allows us to overcome a limit of previous research, namely the use of different methodologies that may have contributed to the contradictory findings in the existing literature.

We will first present four within-language studies in which Italian and German participants were asked to identify the SO of gay and heterosexual speakers (1A and 1B using dichotomous measures and 2A and 2B using continuous measures). Subsequently, we present comprehensive acoustic analyses of these studies. The last study reports identification of SO of foreign language speakers.

## Experiment 1A

### Dichotomous categorization via mouse tracking–Italian sample

Across all studies, participants listened to short voice samples and were asked to judge the speakers’ SO. Using speech samples of identical content, but of different language, we conducted experiments both in Italy (Experiments 1A and 2A) and in Germany (Experiments 1B and 2B) and examined whether similar or distinct patterns of voice-based categorization would emerge; we also tested the relation between the acoustic proprieties of the speakers’ vocal signal and listeners’ judgments of SO.

In Experiment 1A and 1B, we employed a dichotomous forced-choice method combined with a new, for this line of research, method: the *mouse tracker*. Here the answers have to be provided within a limited time window by moving the mouse to the left or right upper corners representing in this case the labels “heterosexual” or “homosexual”. Please note that the Italian term “omosessuale” and the German term “homosexuell” are considered evaluatively neutral and inclusive of men and women in these two languages, and can hence be used in the same way for studies on gay and lesbian speakers.Along with the categorical responses, this measure allows one to record mouse trajectories indicative of the certainty or hesitation with which participants reach the final response. Hand movements are supposed to track the real time dynamic of the categorization process [27], with the advantage to observe not only the outcome of the categorization, but also the unfolding of the process itself.

### Method

#### Ethics statement

The research presented in this paper was approved by the University of Trento ethics committee, and was conducted in accordance with the ethical standards laid down in the 1964 Declaration of Helsinki. All participants provided consent before participating.

#### Participants

Thirty students of a middle size university in the north of Italy took part in the experiment in the role of listeners. Two participants were excluded from the analyses: One identified herself as bisexual and the other reported not to be an Italian native speaker. The final sample consisted of 2^8^ heterosexual Italian participants (14 females, *M*_*age*_ = 20.33, *SD* = 0.99).

#### Speakers

All speakers were recruited through the researchers' contacts, through advertisements placed on University bulletin-boards and, in the case of gay speakers, also through LGBT associations. Previous research has used different methodologies in recruiting speakers. In some studies, speakers were aware of the research topic or were explicitly contacted because of their SO [2, 3], in others no explicit reference to SO as the topic under study was made [4, 6]. We decided to follow this second strategy, thus none of our speakers was informed a-priori about the aim of the research, nor was any reference made to SO. Participants were only told that the purpose of the study was to record materials for future studies. To avoid any suspicious, when we contacted speakers through LGBT associations, we told them we were recruiting non-student participants and the easiest way to obtain a representative sample of the population was to contact different cultural associations in town.

Speakers were recorded individually in a quiet room (sampling at 44 kHz, 16 bit resolution, mono). They were invited to read in a natural way 20 experimental sentences written in their native language, and their voice was recorded using PRAAT [28]. Then, they were asked to fill out a questionnaire including, among other scales, demographic information such as gender, age, and SO. This latter information was provided on a scale from 1(exclusively heterosexual) to 7(exclusively homosexual). Speakers who reported a value above the scale midpoint (i.e., 5 or above) were considered self-identified gays, those reporting a value below the midpoint (i.e., 3 or below) were considered self-identified heterosexuals. At the end, speakers were fully debriefed and signed the consent form approving the use of the audio materials.

Twenty speakers were recorded, 10 of whom self-identified as gays and 10 as heterosexuals. Speakers were between 24 and 40 years of age, with no age difference between self-identified heterosexual (*M* = 31.09, *SD* = 4.90) and self-identified gay speakers (*M* = 29.60, *SD* = 4.74) (*t*(18) = 1.18, *p* >.2). They were all Italian native speakers; to ensure little variability among speakers' accents, all of them came from the North-East of Italy (provinces of Verona and Trento).

#### Sentences

Twenty recorded sentences were used as stimuli (see Appendix A). The sentences were constructed in order to have a similar syntactic structure and a neutral content with reference to SO. Stimuli had approximately the same length (5–9 words, mean length = 6.95, *SD* = 0.99).

#### Procedure

Participants were informed that they would have to categorize speakers as gay or heterosexual on the basis of their voice. To do that, following Freeman and colleagues' [29] procedure, we implemented a categorization task using mouse tracker. Participants completed two blocks in which they listened to 20 sentences produced by 20 speakers. Voices were presented in a randomized order. Participants listened to one sentence for each speaker and, within each block, each speaker pronounced a different sentence. Half of the sentences were pronounced by self-identified gay speakers, half by self-identified heterosexual speakers. All sentences (n = 20) recorded for each speaker were used across participants to assure that type of sentence did not influence results.

Participants were tested individually. While listening, they had to categorize the speakers as gay or heterosexual by clicking on one of the two labels (homosexual vs. heterosexual) displayed on the top left and right corners of the screen. Across participants, the label position was counterbalanced. Participants had to move the mouse toward the label with the category they wanted to select and click on it. The initial position of the cursor was at the bottom center of the screen and participants moved it toward one of the two categories in order to make their choices. Participants were instructed to be as fast as they could, with a limited time (3 seconds) to answer. Using MouseTracker software [27], we recorded response accuracy and mouse trajectories. The experimental session was preceded by a short practice. At the end of the task, participants were asked to report their demographic information (gender, age, SO, nationality, and native language) and were then thanked and debriefed.

### Results

First, we tested whether participants were able to accurately recognize the speakers' SO. In this analysis, response accuracy was tested separately for self-identified gay and heterosexual speakers. Then, we compared participants' mouse trajectories to test how the categorization process unfolded. Finally, we looked separately at each speaker to see which speakers were judged consistently as heterosexual or gay.

#### Sexual orientation–Response accuracy

Inspection of the percentages of correct identifications revealed a different pattern for self-identified heterosexual and self-identified gay speakers: The former were correctly categorized in 63% of all cases which differed reliably from chance (i.e., accuracy at 50%), chi-square = 18.71, *p* < .001, whereas the likelihood of correct identification of gay speakers was below chance (39% correct identifications, chi-square = 14.68, *p* <.001). Thus, whereas self-identified heterosexuals were correctly categorized above chance, self-identified gays were for the most part wrongly categorized as heterosexuals (see S1 Table for statistics for each speaker). As evident from the percentages, the better recognition of heterosexual than homosexual speakers is almost entirely due to a criterion shift. This interpretation is also confirmed by a signal detection analysis that allows us to discriminate between the accurate responses (hit) and the incorrect responses (false alarms). Using this approach we took in account the general tendency to categorize speakers as straight, the *response bias*, and the difficulty in distinguishing between hits and false alarms, namely the *sensitivity* or *discriminability* index [30]. In our study we found a very low discriminability index (*d’* = .054) and a very strong response bias (*β* = .99).

#### Response trajectories

Ample mouse trajectories are generally interpreted as an index of hesitation in providing responses, whereas relatively straight lines suggest a high degree of certainty. To understand how our participants provide responses, we compared the hand movements for correct and for incorrect responses for both self-identified gay and heterosexual speakers. For the analyses, trajectory coordinates were re-scaled into a standard coordinate space (to-left = [1,1.5] and bottom-right = [1,1.5]) and were remapped rightward to allow direct comparisons [27]. The coordinates of the trajectories were time normalized by re-sampling the original vector into 101 time-steps using linear interpolation to average across trials.

#### Spatial attraction–Correct vs. incorrect responses for heterosexual vs. gay speakers

**Fig 1:**
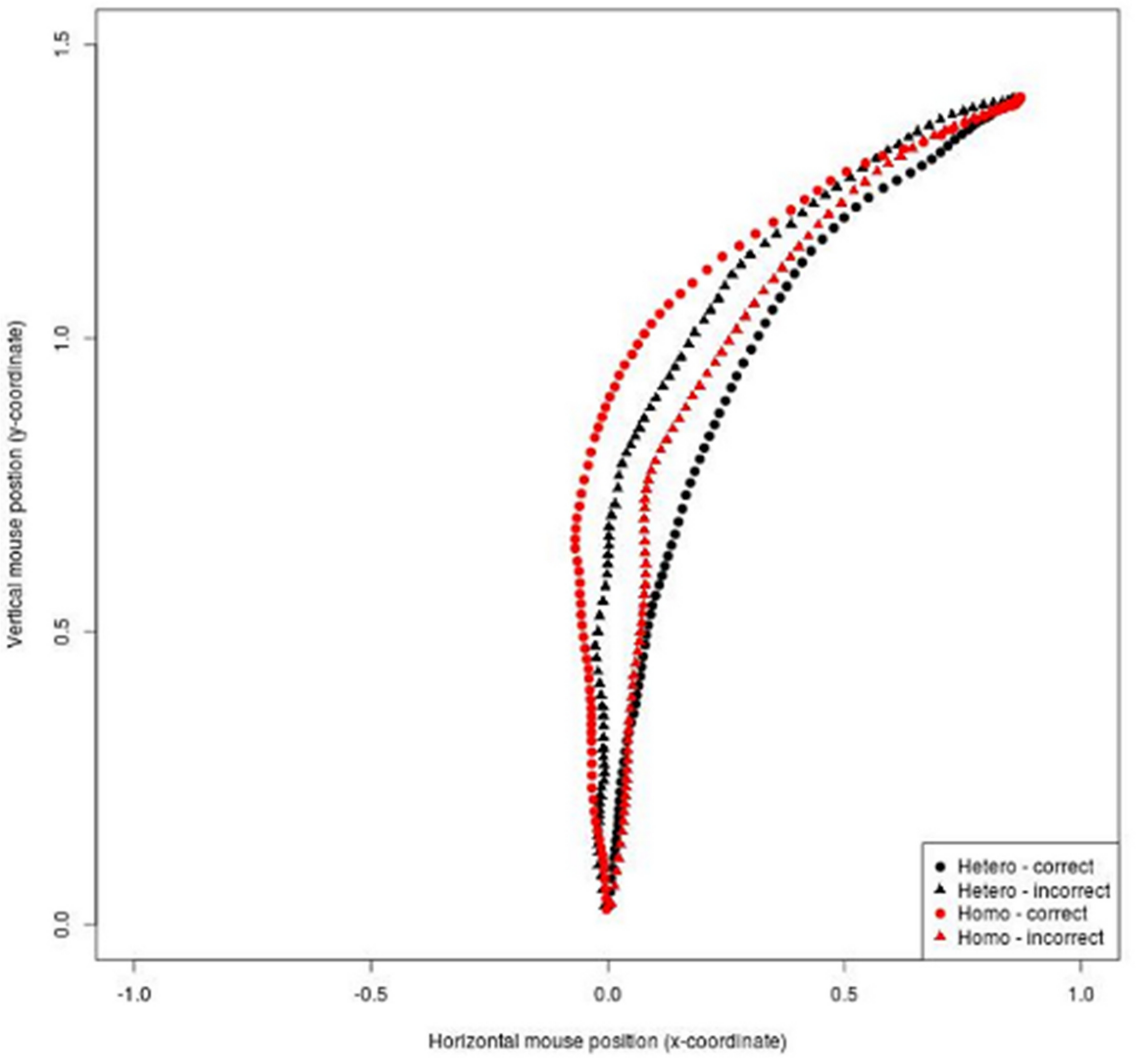
Experiment 1A (Italians): Mean mouse trajectories for correct and incorrect responses for heterosexual and gay speakers.

As an index of the mouse attraction toward the category, we computed the Area Under Curve (AUC; [27]), which is the area between the observed trajectory and an idealized straight-line trajectory connecting the starting point of the movement with the clicked label. Both correct and incorrect responses for heterosexual and gay voices were considered. Mean trajectories are reported in Fig 1.

A mixed-effects model with AUC as dependent variable and Speakers (heterosexual vs. gay) and Response (correct vs. incorrect) as predictor was performed. Participants and stimuli were treated as random factors. The models were fitted using the *lmer* function (lmerTest package version 1.0) in R software (Version 3.1.0).

The model showed a significant interaction between Speakers and Response (*β* = 1.02, st. err. = 0.21, *t* = 4.68, *p* <.001). Further inspection of the interaction showed that, when correctly categorized, the AUC for heterosexual speakers (*M* = .95, *SD* = .68) was smaller than the one for gay speakers (*M* = 1.53, *SD* = 1.11, *p* = .001). The opposite tendency emerged for the incorrect responses: The AUC was larger for heterosexual (*M* = 1.41, *SD* = 1.21) than for gay speakers (*M* = .98, *SD* = .71; *p <* .001). Also, whereas AUC for correct responses was smaller than for incorrect responses in the case of heterosexuals speakers (*p* = .001), the contrary emerged for gay speakers. In this case incorrect answers showed a smaller AUC than the correct ones (*p <* .001). These results suggest that when participants categorized heterosexual speakers correctly they did it without uncertainty, whereas when they categorized gay speakers correctly they were somehow attracted by the incorrect choice (i.e., heterosexual), suggesting that they experienced ambivalence.

A further analysis was run to ascertain that the difference between trajectories in the different conditions was due to a continuous attraction of all the trials toward the opposite category, and not to the presence of a subpopulation of trials in which participants made discrete movements toward the unselected option while, at a certain point, reversing course toward the correct choice. To address the issue the bimodality coefficient (b) for the AUC distribution was calculated (for technical details on the formula, see [27]); if the coefficient was larger than .55, the distribution is considered bimodal. In the case of correct answers, both the coefficient for the AUC-distribution in the heterosexual condition (b = 0.49) and the coefficient for the AUC-distribution in the gay condition (b = 0.36) did not exceed the critical value, so we can reject the bimodality hypothesis. The same was true for the incorrect answers (for heterosexual speakers: b = .37; for gay speakers: b = .43)

Together, the mouse tracking data show that participants were least hesitant in providing responses when correctly identifying heterosexual speakers, but they experienced the highest degree of hesitation when correctly identifying gay speakers.

#### Perceived sexual orientation

**Fig 2:**
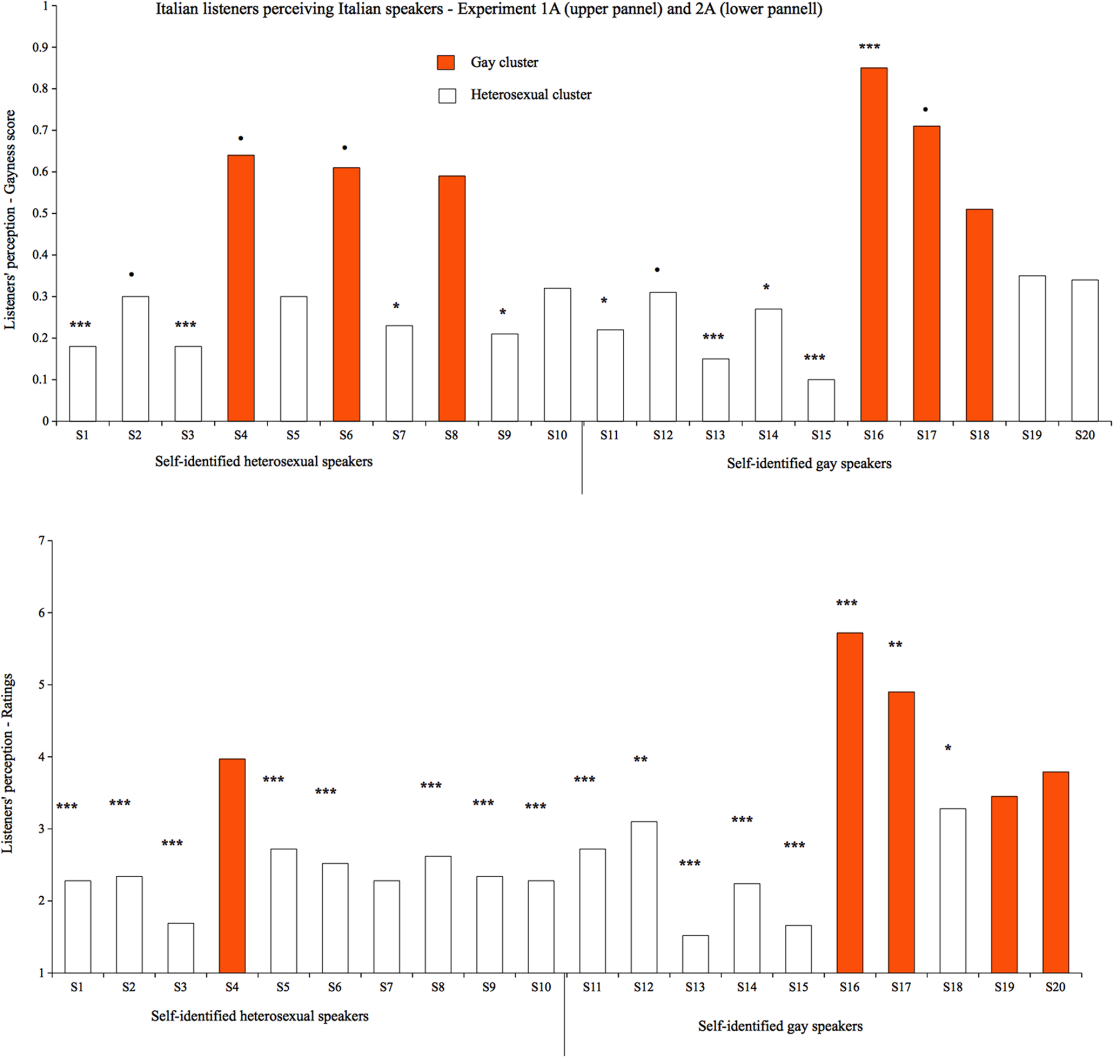
Italian listeners & Italian speakers. **Listeners’ perception in terms of gayness score (2A) and ratings (2B).** Different colors indicate the two clusters based on listeners' perception. Stars (and points) indicate speakers that were significantly perceived either as heterosexual or gay (. < .1; * <.05; ** <.01; *** <.001). Higher values of the y-axis indicate perceived gayity, lower values perceived heterosexuality; .5 represents chance level.

Although participants showed difficulties in correctly categorizing speakers' SO, a look at their responses indicated that they did not categorize speakers in a random fashion. To test this, we calculated the proportion of correct responses for each speaker and compared it to chance level (i.e., accuracy at .5). Regardless of the speakers' self-identified SO, we found that some speakers were categorized as heterosexual and others as gay and, for many of these, this was different from chance. Four of the 10 heterosexual speakers were consistently recognized as heterosexual and none was misidentified as gay. Among the gay speakers only 1 was consistently identified as gay, whereas 4 were misidentified as heterosexual (Fig 2).

Although agreement between participants' judgments and speakers' self-categorizations was low, there was a considerable agreement among the judgments provided by participants. We therefore decided not to consider participants' responses in terms of correctness but to focus on how they perceived the speakers' SO. We thus re-coded all speakers as being *perceived* as heterosexual or gay by calculating a *gayness rating*, ranging from 0 to 1 and defined as the proportion of times the speaker was categorized as gay. The higher the gayness score, the higher the likelihood that the speaker is perceived as gay. To test whether listeners coherently group some speakers on the basis of perceived (not self-identified) SO, a k-means cluster analysis on the gayness values was performed setting k = 2. The analysis revealed that speakers could be grouped in 2 clusters of 14 heterosexual-sounding speakers (gayness rating value of .24) and 6 gay-sounding speakers (gayness rating mean value of .65), respectively; the assumption of two clusters fit quite well with the observed data, as shown by the fact that this division accounted for 83% of data variability. The decision to have two clusters was motivated by the following reasoning: First, we were looking at two distinct groups based on sexual orientation such as sounding gay and sounding heterosexual speakers. Second, there is no numerical support for a three-cluster grouping, as shown by the fact that the imposition of three clusters instead of two produced only very small increase in explained variance (only 6%). Gayness values and clusters are reported in Fig 2.

These results show that listeners consistently categorized some speakers as gays or as heterosexuals on the basis of what they sounded like. Note that this is largely independent of the SO indicated by the speakers themselves. Moreover, the distribution of gay- and heterosexual-sounding speakers was not coherent with the real (50:50) distribution of voices. The majority of the speakers were perceived as sounding heterosexual whereas only a minority was considered as sounding gay.

## Experiment 1B

### Dichotomous categorization via mouse tracking–German sample

Experiment 1B was identical to the previous one, except for 3 aspects: 1) it was run in Germany and hence in German language. 2) It included 12 (rather than 20) speakers, half of which self-identified gays, half self-identified heterosexuals. 3) Instructions were varied such that half of the participants were informed about the fact that half of the speakers were heterosexual and half gay. Each of these differences will be explained below. All other aspects of the materials, procedure and analyses were identical to Experiment 1A.

### Method

#### Participants

Forty-eight university students took part in the experiment in the role of listeners. Four participants were excluded from analyses because they identified as gays or bisexuals. The final sample consisted of 44 heterosexual participants (22 females, *M*_*age*_ = 26.95, *SD* = 4.97) who were German (n = 39) or had lived in Germany for more than 7 years (n = 5). Note that analysis excluding those participants who self-identified as non-German but regularly lived in Germany for a consistent period of time did showed the same pattern of results.

#### Speakers

Twelve speakers were recorded, including six self-identified gays and 6 self-identified heterosexuals. Their age varied between 20 and 40, however, no age difference emerged between self-identified gay (*M* = 28.50, *SD* = 3.02) and self-identified heterosexual speakers (*M* = 28.17, *SD* = 7.13), *t*(10) = .10, *p* = .92. All speakers lived in North Rhine-Westphalia and spoke standard German.

#### Sentences

Twelve recorded sentences were used as stimuli. These were the first sentences from the list used in Study 1A. They were all translated from Italian into German and had a similar length (5–10 words, mean length = 7.00, *SD* = 1.48).

#### Instructions

Study 1A had revealed that participants were rather inaccurate in their attempt to identify the SO on the basis of voice alone. This inaccuracy may derive from the fact that our voice sample contained 50% self-identified gay and 50% self-identified heterosexual speakers, whereas participants may have assumed a different baseline, building on their knowledge that gays constitute a minority in the general population. This may easily explain why they were particularly likely to falsely classify gay speakers as heterosexual. To test this possibility, in Study 1B we manipulated the type of instruction participants received. As in Study 1A, participants were asked to listen to the speakers and to judge their SO. However, at the beginning of the task, half of participants were informed that they would listen to 6 gay and 6 heterosexual speakers, whereas the other half did not receive this additional information. If the inaccuracy in Study 1 had been caused by an unexpected distribution, then this difficulty should disappear when people know the actual (50:50) distribution. Contrary to this logic, no difference was found between informed and uninformed participants. Regardless of whether or not participants knew the actual distribution of gay and heterosexual speakers, participants’ ability to identify gay voices did not differ from chances (no info: 46% and 50:50 info: 50%), whereas heterosexual voices were identified reliably above chance in both conditions (no info: 67% and 50:50 info: 64%). Hence, this variable will be no further discussed. Importantly, however, the lack of a difference between the two conditions proves that differential baseline assumptions cannot account for the inaccuracy in participants’ responses.

### Results

#### Sexual orientation–Response accuracy

On average, self-identified heterosexual speakers were correctly recognized in the majority of cases (66%), whereas self-identified gay speakers were not (48% of correct identifications). Correct identifications exceeded chance for self-identified heterosexual (chi-square = 24.60, *p* < .001), but not for self-identified gay speakers (chi-square = .33, *p* = .56). This pattern suggests a clear difficulty in identifying SO of gay speakers (see S1 Table for statistics for each speaker), which is mainly due to the overwhelming tendency to identify our male speakers as heterosexual. In fact, applying a signal detection approach, participants showed a strong response bias (*β* = 1), but a low discriminability index (*d’* = .20). As in Experiment 1A, we proceeded to examine first the participants' mouse trajectories, and then participants' perceived SO for each speaker.

#### Response trajectories

The same analyses as in Study 1A were conducted comparing correct and incorrect responses for self-identified heterosexual and gay speakers.

#### Spatial attraction–Correct vs. incorrect recognition of heterosexual vs. gay speakers

**Fig 3:**
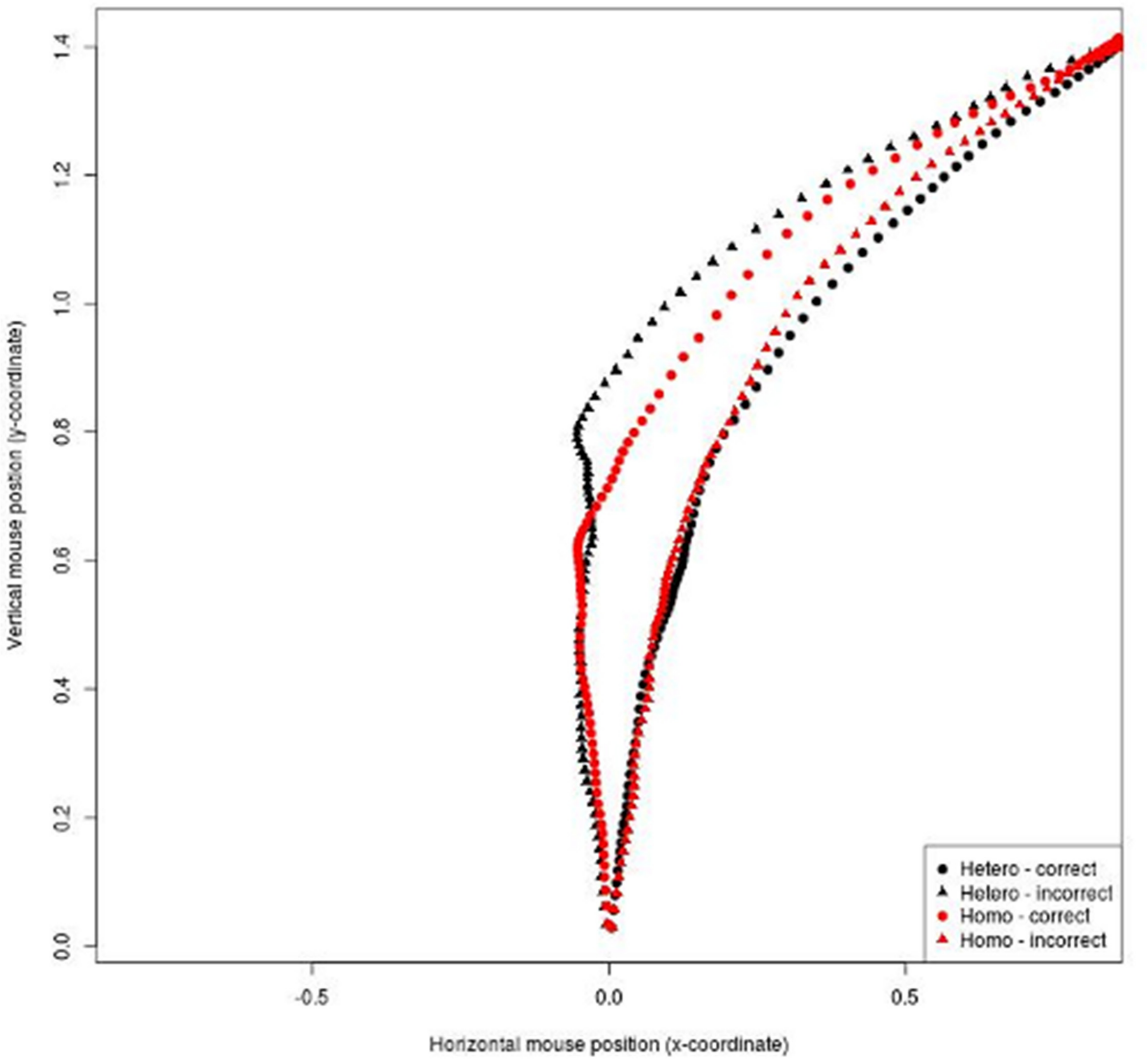
Experiment 1B (Germans): Mean mouse trajectories for correct and incorrect responses for heterosexual and gay speakers.

Both correct and incorrect responses for heterosexual and gay voices were considered using the AUC as index of the mouse attraction toward the category. Mean trajectories for conditions are reported in Fig 3.

A mixed effects model with AUC as dependent variable and Speakers (heterosexual vs. gay) and Response (correct vs. incorrect) as predictors was run. Participants and items were treated as random factors. The model showed a significant interaction between Speakers and Response (β = 0.76, SE. = 0.19, *t* = 4.01, *p* <.001). Further inspection of the interaction showed that AUC was smaller for correct identifications of heterosexual (*M* = .79, *SD* = .68) than gay speakers (*M* = 1.15, *SD* = 1.04; *p* = .002), whereas it was larger for incorrect identifications of heterosexual (*M* = 1.36, *SD* = 1.17) than of gay speakers (*M* = .90, *SD* = 1.12; *p* = .006). Moreover, for heterosexual speakers the AUC was smaller for the correct than for the incorrect responses (*p <* .001), whereas for correct and incorrect responses in categorization of gay speakers the difference only approached significance (*p* = .07). To ascertain the reliability of the results, we calculated the bimodality coefficient (b) for the two AUC distributions. For correct answers, bimodality can be excluded given that neither the coefficient for heterosexuals (b = .47) nor that for gays (b = .44) exceeded the threshold of .55. As no difference emerged for the incorrect responses, no bimodality coefficient was examined.

Taken together, findings were similar to those obtained on Italian participants. Again, participants showed greater hesitation when providing incorrect (rather than correct) responses. They also showed greater hesitation when misidentifying heterosexual speakers as gay than when correctly identifying them as heterosexual. This time, no reliable differences emerged for gay speakers.

#### Perceived sexual orientation

**Fig 4:**
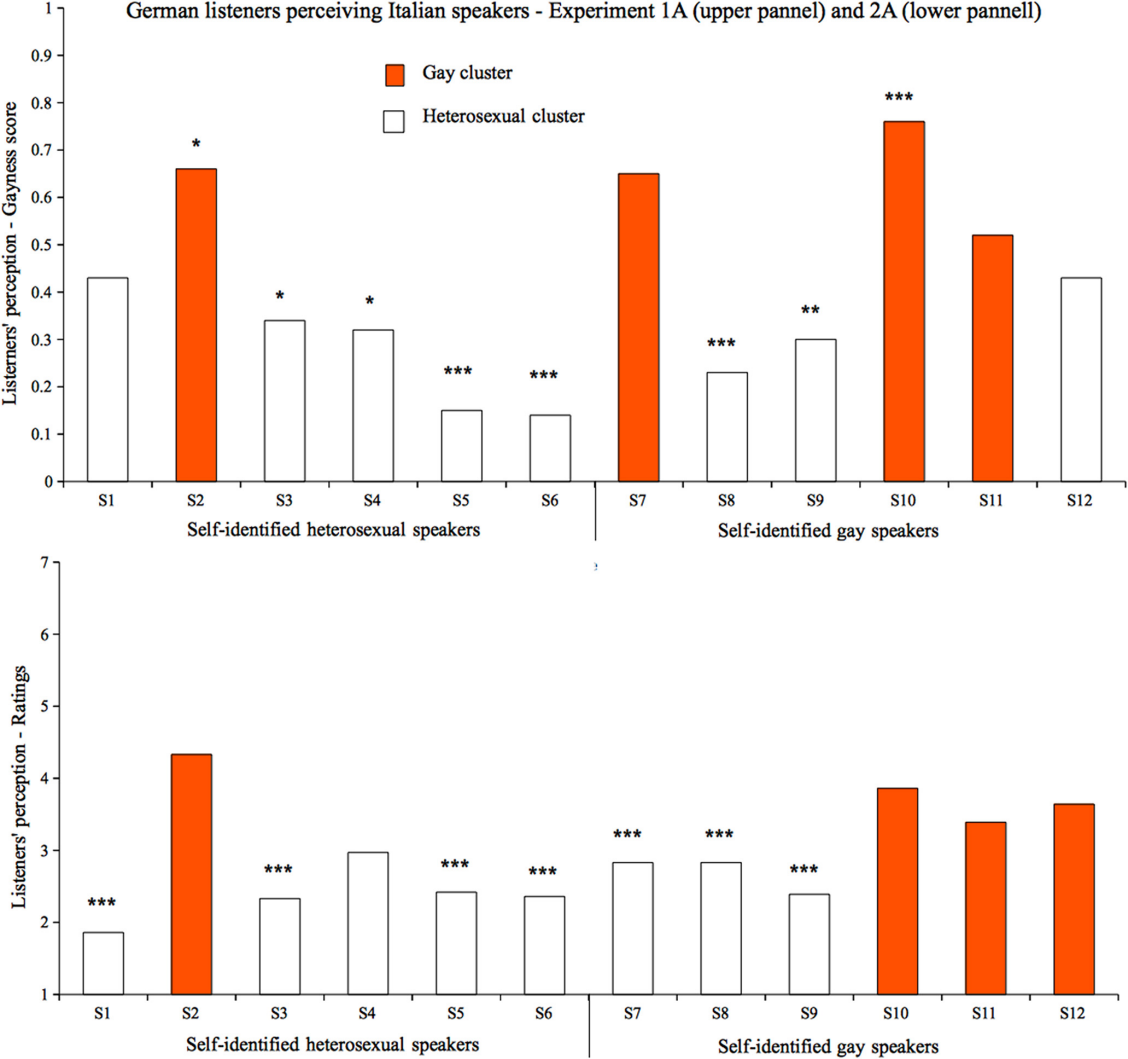
German listeners & German speakers. **Listeners’ perception in terms of gaynessscore (4A) and ratings (4B).** Different colors indicate the two clusters based on listeners’ perception. Stars (and points) indicate speakers that were significantly perceived either as heterosexual or gay (. < .1; * <.05; ** <.01; *** <.001). Higher values of the y-axis indicate perceived gayity, lower values perceived heterosexuality; .5 represents chance level.

Regardless of the self-identified SO, some speakers were more likely to be categorized as heterosexual and others as gay (Fig 4) and, for most of the speakers, this was different from chance. Comparing responses to chance, 4 of the heterosexual speakers were correctly identified as heterosexual and one was incorrectly perceived as gay. Of the gay speakers, two were misidentified as heterosexual and only one was correctly identified as gay.

We again calculated the gayness score and tested whether listeners categorized speakers in two groups (i.e., gay- vs. heterosexual-sounding) depending on how voices were perceived. A K-means cluster analysis on the gayness values was performed setting k = 2. We found that a 2 clusters solution explained 74.2% of the variance. Eight speakers were included in the first cluster (mean value of .29) whereas the remaining 4 were included in the second cluster (mean value of .65). Gayness values and clusters for each speaker are reported in Fig 4.

## Experiment 2A

### Within-language judgments on continuous scales–Italian participants

Our first set of studies showed that Italian and German listeners were not very accurate in judging SO of speakers of their own native language. They generally tended to categorize speakers as heterosexuals and in both samples only one gay speaker was reliably identified as gay. Even when they correctly categorized the self-identified gay speakers, they tended to be attracted towards the opposite response (“heterosexuals”), suggesting a certain degree of uncertainty, at least in the Italian sample. Although indicative of inaccuracy of the voice-based categorization of SO process, this may be a function of the forced choice format that, combined with a strict time limit, may have made the task difficult. In studies 2A and 2B we therefore asked participants to rate speakers' SO on a scale that allowed them to modulate their answers without time limit (see [3], for a similar procedure). Thus, listeners were given the possibility to mold their judgments without requiring a dichotomous decision and without imposing a response time limit. An additional advantage of this method is that speakers’ self-definitions and listeners’ perception of SO were assessed on identical scales that allow a direct comparison between the two.

### Method

#### Participants

Thirty university students (15 female, *M*_*age*_ = 20.97, *SD* = 2.37) took part in the experiment in the role of listeners. All participants were Italian native speakers.

#### Materials

Voice samples and sentences were the same as in Experiments 1A.

#### Procedure

Participants were tested individually. In the experiment they performed a computer task, in which, for each trial, participants listened to a spoken sentence through headphones. In one block, participants had to judge the speaker's SO on a scale from 1 (*completely heterosexual*) to 7 (*completely homosexual*). In another block, among other dimensions, participants evaluated the speakers' masculinity on a scale from 1 (completely masculine) to 7 (completely feminine). As this research aims at studying the perception of sexual orientation, we do not further consider other dimensions (e.g., age) that have been measured (see also S2 Table).

### Results

Analogous to the procedure in the previous experiments, we first tested whether participants were generally able to distinguish heterosexual from gay speakers. Then, we tested how listeners perceived speakers' SO by verifying whether there was any consistency in the way participants judged the voices.

#### Sexual orientation–Response correctness

To test for accuracy, different analyses were performed. First, we tested the relative accuracy of the participants’ guesses. To do so, we adopted a method that is roughly based on Cadinu and Rothbart’s [31] “within-participants correlations” methodology (see also [32]). We calculated for each participant the correlation between his/her ratings (perceived SO) and the speakers’ self-identified orientation. Different from Cadinu and collaborators [31, 32], we used Spearman correlations because the self-reported SO was not distributed normally across speakers. The within-participants correlations were then z-tansformed and treated as dependent variables and subjected to a one-sample t-test. Positive correlations that deviated reliably from zero suggest that the participants’ ratings were above chance. The average within-participants correlations (*M* = .158, *SD*. = .21) was indeed positive and deviated reliably from chance, *t*(28) = 4.06, *p* < 001, suggesting that participants were to some degree able to identify the speakers’ SO. At the same time, the small entity of the correlation suggests that the ability to guess the speakers’ SO, though slightly above chance, was all but perfect.

Second, to better understand how speakers were evaluated we compared ratings for the group of heterosexual and gay speakers. Overall, self-identified gay speakers (*M* = 3.24, *SD* = 1.33) obtained higher ratings (meaning greater perceived homosexuality) than self-identified heterosexual speakers (*M* = 2.50, *SD* = .58), (*t* (29) = 6.39, *p* < .001), suggesting, again, that participants were, to some degree, able to distinguish the two groups from voice alone. However, both means were reliably below the scale mid-point (one-sample *t*s > 4.50 and *p*s < .001), showing that both self-identified heterosexual and self-identified gay speakers tended to be rated as heterosexual. These findings demonstrate again that participants categorized speakers mainly as heterosexual. Moreover, there was high variability on accuracy ratings across speakers (see below, Fig 1 and S3 Table for statistics for each speaker).

#### Perceived sexual orientation

In order to test whether each speaker had received consistent ratings across participants regardless of his actual SO, we looked at each speaker separately, using perceived SO as dependent variable and comparing the mean ratings to the neutral scale midpoint.

The data reported in the lower portion of Fig 2 show that the majority of the speakers were perceived as heterosexual (including all but one heterosexual speakers and six out of ten gay speakers). Only two speakers were consistently perceived as gay and both of these were self-identified gays.

Moreover, as in Experiment 1A, we looked at whether speakers could be divided into two groups (heterosexual-sounding vs. gay-sounding) on the basis of participants’ judgment. To this aim, we ran a k-means cluster analysis on the mean ratings setting k = 2. The two clusters were identified as follows: The first group was composed of 15 speakers, with a mean value of 2.3; the second group was composed of 5 speakers (see Fig 2), with a mean value of 4.3; the two clusters accounted for 68% of data variability. Although the imposition of three clusters would produce an increase of 19% of the explained variance, we were still looking to distinguish two groups of speakers as we also asked participants to do so. Note that the pattern shown by the cluster analysis was similar to that of Experiment 1A. Excluding those speakers that were judged at chance level in both experiments, 15 out of 18 speakers were collocated in the same clusters as in Experiment 1A.

#### Correlation between perceived sexual orientation and masculinity

A correlation was run to explore the link between perceived SO and masculinity. The analysis revealed a strong correlation between the two measures (Spearman correlation, *ρ* = -.59, *p* = .009): The more the speakers were perceived as masculine, the less they were judged as gay.

## Experiment 2B

### Within-language judgments on continuous scales–German participants

This experiment was identical to study 1B. However, in this case the response format consisted of a 7 point Likert-type scale like in Study 2A and participants were asked to evaluate both speaker's SO and masculinity.

### Method

#### Participants

Thirty-six university students took part in the experiment in the role of listeners. Four participants were excluded from analyses because they identified as gays or bisexuals. The final sample consisted of 32 heterosexual participants (16 female, *M*_*age*_ = 25.75, *SD* = 6.96) who were German or spoke perfectly German.

### Results

#### Sexual orientation–Response correctness

As in Study 2A, we correlated each participant’s SO ratings with the speakers’ self-reported SO and used the resulting within-participants correlations as dependent variables. Again, accuracy was above chance but of very small magnitude (*M =* .127, *SD* = .33), one-sample *t*(34) = 2.31, *p* = .027. Looking at the mean SO ratings, data suggest that on average, self-identified heterosexual speakers (*M* = 2.71, *SD* = .84) were judged as more heterosexual than gay speakers (*M* = 3.12, *SD* = 1.11), (*t* (31) = 2.22, *p* = .03), suggesting that, to some degree, participants were able to grasp differences in SO. However, as in Experiment 2A both means were significantly below the mid-point of the scale (both *t*s > 4.76, *p*s <.001), attesting to the fact that participants tended to perceive speakers as heterosexual (see S2 Table for statistics for each speaker).

#### Perceived sexual orientation

As illustrated in the lower portion of Fig 4, participants had difficulties in detecting gay speakers: The majority of speakers were perceived as sounding heterosexual. Comparing mean ratings to the scale midpoint, of the 6 heterosexual speakers, 5 were correctly perceived as heterosexual and none was falsely classified as gay. Of the 6 gay speakers, only 1 was reliably identified as gay and 3 were falsely classified as heterosexual. Ratings for the remaining speakers did not differ from the scale midpoint.

We further analyzed whether speakers could be divided into two groups (i.e., gay-sounding-vs. heterosexual-sounding) according to listeners' perception, regardless of their self-identified SO. The k-means cluster analysis on the mean rated values (and setting k = 2) yielded two clusters assigning 8 speakers to the first (mean value of 2.50) and 4 speakers to the second cluster (mean value of 3.80). The two clusters accounted for 77% of data variability. The analyses suggest that some speakers were strongly identified as heterosexual, whereas other speakers tended to be perceived as less heterosexual and were located around the scale midpoint. The results of the cluster analysis are quite similar to those reported in Experiment 1B: Ten out of the 12 speakers were located in the same cluster in the two experiments.

#### Correlation between sexual orientation and masculinity

As for Italians, we explored the relation between the perception of speakers’ SO and masculinity. Analysis revealed no reliable relation between perceived SO and masculinity (*r* = -.46, *p* = .12), although the magnitude of the correlation was only slightly smaller than that of the Italian sample.

## Acoustic Analyses

All four studies suggest that people are rather inaccurate in identifying the SO of speakers on the basis of voice alone and that heterosexuality serves as a default option even when the actual 50:50 distribution is known. Interestingly, however, people do not respond in a random fashion but associate certain voices with heterosexuality and others with gayness. The question therefore arises whether voices carry information, independent of the self-defined SO of the speaker. What makes some voices appear gay and others heterosexual? To answer this research question, we explored the relation between the participants' perception of speakers' SO and a sub-set of acoustic properties of the speech signal, with the aim to identify which acoustic cues Italian and German participants exploited to perform their judgments. Moreover, since, at least for Italian, the perception of speaker's SO seems to be related to that of speaker's masculinity, we also explored the relation between the latter and the speech signal.

In the acoustic analyses we focused mainly on the segmental level of speech (vowels and consonants) but also included vowel duration, which is a suprasegmental feature. Hence, we selected those acoustic features that had already been reported in the literature as (potentially) relevant for the recognition of speakers' SO (e.g., [3–6]). Note that the two kinds of acoustic measures provide distinct information, as the frequency parameters are related to (and depend on) the physical properties of the oral cavity–and are thus stable for each speaker–whereas the duration measures depend more on the speaking style and may therefore vary according to the speaker’s intentions.

We considered the following features for vowels: duration (in ms), F0, F1, and F2 (all Fs in Hz). For the sibilant fricative /s/ we considered: duration (in ms), center of gravity (in Hz), skewness, and kurtosis. Acoustic measures were made using the PRAAT software [28]. The onset and offset of each phoneme of interest in each word was marked in PRAAT by a coder. All acoustic analyses were done automatically in PRAAT using custom-written scripts, which made reference to these labels. Formants were measured at vowel midpoint. Acoustic measures were extracted by a sub-set of all recorded materials (20 tokens for each sound in Italian and German, respectively; Italian vowels: /a/, /e/, /i/, /o/, /u/; German vowels: /a/, /a:/, /e:/, /ε/, /ε:/, /i/, /I/, /i:/, /o/, /o:/, /u:/; only stressed tokens were selected). Finally, we considered the speaking rate (measured as the ratio between total sentence duration and the number of syllables in the sentence).

To explore whether and what acoustic features participants related to heterosexual- vs. gay-sounding voice, correlation analyses were run between the listeners' judgments and all acoustic measures. Then, to explore whether the same cues are also related to the masculine- vs. feminine-sounding voices, the same acoustic analyses were run between acoustic cues and ratings of masculinity. Pearson correlations were adopted when both measures in the correlation had a normal distribution; in all the other cases Spearman correlations were run. Finally, we tested whether speakers perceived as gays speak differently from those perceived as heterosexual. Hence, we considered heterosexual- and gay-sounding speakers (based on cluster analyses reported above) and compared whether the two groups of speakers differ on acoustic cues. Analyses were performed only for those acoustic features that were found to be significantly related with listeners' ratings.

### Italians—Experiment 1A (dichotomous choice)

Since the duration measures of all vowels were highly correlated with each other, we calculated a vowel duration index as a global measure of all vowel durations (computed by averaging the duration of all vowels). The new measure was highly correlated with all single measures (/a/: *r =* .93; /e/: *r =* .94; /i/: *r =* .98; /o/: *r =* .94; /u/: *r =* .68) and can therefore be considered a valid compound measure of vowel duration. The mean vowel duration, however, did not strongly correlate with speaking rate (*r =* .53) and /s/ duration (*r =* .48), suggesting that these are better considered separately. Table 1 reports only the significant correlations between the gayness score on one side and the frequency parameters and the duration measures on the other (for the full list of correlations for this and the following experiments, see S4 Table).

**Table 1:** Italians–Significant correlations between acoustic cues and participants' judgments of Experiment 1A, 2A, and self-reported speakers' ratings.

	Sexual Orientation						Measure of Masculinity	
	Experiment 1A		Experiment 2A		Italian Speakers		Experiment 2A	
Acoustic measures	Correlation with Gayness rating	p	Correlation with listeners' rating	p	Correlation with self rating	p	Correlation with listeners' rating	p
Vowel F1								
vowel /i/	—	—	ρ = .48	*	ρ = .46	*	—	—
vowel /u/	—	—	—	—	ρ = .51	*	—	—
Vowel F2								
vowel /a/	ρ = .55	*	—	—	—	—	—	—
vowel /e/	ρ = .67	**	—	—	—	—	—	—
/s/ center of gravity	ρ = .57	*	ρ = .52	*	—	—	r = .46	*
/s/ skewness	—	—	—	—	—	—	r = .46	*
Duration measures								
Speaking rate	ρ = .71	***	ρ = .53	*	—	—	—	—
Mean vowel duration	ρ = .54	*	ρ = .46	*	ρ = .46	*	—	—
/s/ duration	ρ = .44	*	—	—	—	—	—	-

Note: Positive correlations indicate that the vocal cue is associated with greater perceived gayity. If one of the studies produced significant correlations for a given cue, we also report in italics any correlations in the other study up to p = .2; *p* is the p value:. .2;

*<.05;

**<.01;

***<.001.

Speakers with higher values of gayness (thus, more likely to be perceived as gay) tended to speak slower, and to produce longer vowels than speakers with lower values of gayness. To ascertain whether both speaking rate and vowel duration contribute to the perception of SO a regression analysis was run with duration of vowels, duration of /s/, and speaking rate as predictors. Since such measures were correlated to each other^7^, residuals were entered as predictors in the regression analysis (see, e.g., [33, 34]). Results show that only speaking rate (β = -1.12, st. err. = .11, t = -2.99, p = .009)significantly predicted listeners’ ratings of speakers’ SO (mean vowel duration: t = 1.09, p >.2; /s/ duration: t < 1, p >.7). Moreover, a higher gayness was associated with a higher mean frequencies of /s/, and a higher F2 for the vowels /a/ and /e/.

We then tested whether clusters of speakers perceived as heterosexual vs. gay show differences in those acoustic features that significantly correlated with listeners' ratings. The analysis shows that speakers in the two groups differed only with respect to the center of gravity (Mann-Whitney test, *W* = 73, *p* = .01; all other *p*s >.9).

### Italians—Experiment 2A, sexual orientation (Likert scale)

The same acoustic features tested in Experiment 1A were correlated with the participants’ mean ratings. Significant correlations are reported in Table 1. The analysis shows that speakers perceived as more gay-sounding tended to speak slower, and to produce longer vowels than speakers with lower mean rates (i.e., more heterosexual speakers). The regression analysis with the listeners' rating as dependent variable and the duration of vowels and speaking rate as predictors showed that speaking rate (β = -1.13, st. err. = .51, t = -3.38, p = .004), but not mean vowel duration (β = .73, st. err. = .62, t = 2.21, p = .04) predict the perceived SO. Moreover, speakers perceived as more gay had higher mean frequencies of /s/, and a higher F1 for vowels /i/.

As for Experiment 1A, speakers were divided in two groups (perceived gays and perceived heterosexual) according to the cluster analysis reported earlier. The comparison of the acoustic features of speakers in the two groups shows that they only differed in terms of center of gravity (Mann-Whitney test, *W* = 62, *p* = .03; all other *p*s >.9).

In both Experiments 1A and 2A, participants used speaking rate, mean vowel duration, and /s/ center of gravity to perform their judgment; some of these cues were consistently used in the two studies, suggesting that the way of speaking may be related to the perception of speakers’ SO. The conclusion is further strengthened by the comparison of the speech features of speakers categorized as members of different groups: In both experiments, the /s/ center of gravity is a discriminant for the categorization of a speaker as gay (in case of higher mean frequencies of /s/) or heterosexual (in case of lower mean frequencies of /s/).

### Italians—Experiment 2A, masculinity (Likert scale)

The correlation analyses between acoustic cues and participants’ mean rating show that participants tended to rate speakers as more feminine the higher their mean frequencies of /s/ and the higher their values of /s/ skewness (significant correlation in Table 1). Interestingly, the mean frequencies of /s/ was found to be related also to the judgment of speakers' SO.

### German—Experiment 1B (dichotomous choice)

The same acoustic features and correlation analyses of Experiment 1A were run with the German sample. Significant correlations are reported in Table 2. Our findings, again, suggest that speakers’ judgments are driven by specific acoustic features. In particular, speakers who produced longer sounds tended to be perceived as more gay-sounding; moreover, the gayness score was associated with a higher F2 in some frontal vowels.

**Table 2:** Germans–Significant correlations between acoustic cues and participants' judgments of Experiment 1B, 2B, and self-reported speakers' ratings.

	Sexual Orientation						Measure of Masculinity	
	Experiment 1B		Experiment 2B		Italian Speaker		Experiment 2B	
Acoustic measures	Correlation with Gayness rating	p	Correlation with listeners' rating	p	Correlation with self- rating	p	Correlation with listeners' rating	p
Vowel F0								
vowel /a/	—	—	—	—	—	—	r = -.77	**
vowel /a:/	—	—	—	—	—	—	r = -.80	**
vowel /e/	—	—	—	—	—	—	r = -76.	*
vowel /i/	—	—	—	—	—	—	r = -60	*
vowel /i:/	—	—	—	—	—	—	r = -66	*
vowel /o/	—	—	—	—	—	—	r = -74	*
vowel /u/	—	—	—	—	—	—	r = -77	*
Vowel F1								
vowel /a/	—	—	—	—	ρ = .66	*	—	—
vowel /a:/	—	—	—	—	ρ = .75	*	—	—
vowel /ɛ/	—	—	—	—	ρ = .80	***	—	—
Vowel F2								
vowel /a/	r = .44	.	r = .62	*	—	—	—	—
vowel /a:/	—	—	—	—	—	—	—	—
vowel /e:/	r = .59	*	r = .48	.	—	—	—	—
vowel /ɛ/	r = .70	*	r = .73	**	—	—	—	—
vowel /ɪ/	r = .62	*	r = .63	*	—	—	—	—
Duration measures								
vowel /e:/	ρ = .72	*	ρ = .54	.	—	—	—	—
vowel /i:/	r = .60	*	r = .44	.	—	—	—	—
vowel /u:/	r = .67	*	r = .57	.	—	—	—	—
/s/ duration	r = .66	*	—	—	—	—	—	—

Note: Positive correlations indicate that the vocal cue is associated with greater perceived gayity. If one of the studies produced significant correlations for a given cue, we also report in italics any correlations in the other study up to p = .2; *p* is the p value:. .2;

*<.05;

**<.01;

***<.001.

To further test how the gayness ratings work, the speakers were divided into two groups (perceived gays and perceived heterosexuals) according to the cluster analysis reported above. Acoustic measures of speakers belonging to the two groups were compared. The analysis shows significant differences for the F2 of /e:/ and /ε/ (Mann-Whitney test, *W* = 4, *p* = .03, and *W* = 3, *p* = .02, respectively; all other *p*s > .09).

### German—Experiment 2B, sexual orientation (Likert scale)

Correlation analyses were run between the listeners' mean ratings and the same acoustic features used in Experiment 1B. Significant correlations are reported in Table 2 (for the full list of correlations, see S3 Table). Speakers who produced higher F2 of some centro-frontal vowels tended to be perceived as more gay-sounding. Also, vowel duration seems to play again some role, as suggested by the correlation between duration of, e.g., /u:/ and listeners ratings. Moreover, the comparison of acoustic measures of speakers belonging to the two cluster identified through the cluster analysis above show significant differences for the F2 of /a/ and /ε/ (Mann-Whitney test, *W* = 28, *p* = .05, and *W* = 28, *p* = .05, respectively; all other *p*s > .1).

The results of Experiments 1B and 2B partly overlap: In both cases, German participants tended to perceive the speakers' homosexuality based on the F2 height of some vowels plus and on durational measures, suggesting that the way of speaking is related to the perception of speakers' SO. Moreover, F2 measures were also found to differentiate the speech of speakers categorized as gay from those categorized as heterosexual, with the former showing higher frequency values than the latter.

### German—Experiment 2B, masculinity (Likert scale)

The correlation analyses between acoustic cues and participants’ mean rating showed that femininity was associated with higher values of vowels F0 (significant correlation in Table 2). There was no overlap between the cues related to masculinity and those related to SO.

To sum up, the correlations between acoustic features and listeners' judgments of SO reveal some within-language consistency: Italian participants perform the categorization using mean vowel duration and speaking rate, plus the center of gravity of /s/; the latter measure clearly differentiates the speakers categorized as heterosexual from those categorized as gay, suggesting that gayness ratings are highly driven by these features. In a different way, Germans base their categorization on some duration measures (e.g., /u:/ duration) and on a phonetic property of some frontal vowels (i.e., F2 of /ε/, /I/, /a/). Among these measures, F2 values seem to be particularly relevant for German listeners, as shown by the fact that speakers in the gay cluster have higher F2 values than those in the heterosexual cluster.

It is worth nothing that in both languages and across experiments, durational measures seem to play an important role in driving listeners' perception, whereas the relation between formant frequencies and listeners' perception seems to be stable only in German. Across experiments, we also found sporadic effects for some acoustic features (e.g., F2 of /a/ in Experiment 1A, but not in 2A; duration of /s/ in Experiment 1B, but not in 2B). Although such complex patterns suggest that participants' judgments are based on multiple criteria, these findings ought to be interpreted with caution, given the limited number of available speakers (20 Italians and 12 Germans) and the non-representative sample of sentences.

### Self-identification of Italian and German speakers

Another issue that it is worth investigating, even though in an exploratory way, is the acoustic cues as a function of actual (self-defined) rather than perceived SO. In order to do so, we looked at the correlations between acoustic cues and speakers' self-identification. In this way we were able to test whether heterosexual and gay speakers show differences in the way they speak, and, if this was the case, to what extent the listeners' perception mirrors speakers' differences.

The same acoustic features as those tested with listeners were used. Note that speakers reported their SO on the same Likert scale that participants used in Experiments 2A and 2B. Significant correlations for Italian and German speakers are reported in Tables 1 and 2, respectively.

Italian speakers who self-identified more strongly as gay were also found to produce longer vowels and higher F1 of some vowels (/i/ and /u/). Differences in terms of F1 were also found for German speakers: the more they self-identified as gay the higher F1 they produced for some vowels (/a/, /a:/ and /ɛ /). Overall, SO of Italian and German speakers did not show the same relations with acoustic cues, suggesting that SO is expressed through distinct, language-specific cues. Moreover, for Italians there is partial correspondence between the acoustic cues related to self-defined and perceived SO, at least in terms of vowel duration. It seems that producing longer vowels and speaking somewhat slower is a feature of gay Italian speakers that is also used by listeners in judging SO from voice. For Germans, no correspondence was found between the acoustic cues related to self-identified and those related to perceived SO.

### Discussion

Together, the four experiments show a straightforward pattern of results: Participants are not particularly accurate in categorizing the SO of the speakers they heard. However, there is converging evidence that listeners made distinctions among speakers, as they consistently categorized some of them as gay and others as heterosexual, even where this perception did not correspond to the speaker’s actual SO. Moreover, as ratings (Study 2A and 2B) and mouse trajectories (Study 1A and 1B) show, listeners seemed to consider heterosexuality as a reference level. Across studies, approximately 2/3 of heterosexual speakers were correctly identified as heterosexual, whereas one was identified as gay. Among gay speakers, less than 20% were identified as such and close to half of the gay speakers were erroneously perceived as heterosexual. Thus, a small group of speakers seemed to deviate from the typical heterosexual-sounding voice and were therefore judged to be gay, whereas the majority of speakers were classified as heterosexual. From a methodological point of view it is interesting to note that similar result patterns emerged when participants were asked to perform a dichotomous choice and when asked to rate speakers on a Likert scale, suggesting that the type of judgment does not modify the perception of speakers' SO.

Interestingly, the results of the experiments show a parallel pattern for Italian and German participants, suggesting that the voice-based categorization process works similarly in these two languages. Across studies and speaker groups, both Italians and Germans correctly identified about 40% and misidentified about 25% of the targets, attesting to the cross-cultural stability of this voice-based categorization process. Also, the acoustic analyses suggest that there are some acoustic features that play a role in both languages, as shown by the fact that both Germans and Italians exploit duration properties of speech to make their judgment (longer values associated with higher likelihood to perceive the speaker as gay). This fact is particularly interesting given that duration is more flexible than other properties (e.g. formant frequencies) and can vary within-speakers. In fact, duration properties may be affected not only by speaker intentions, but also by social variables (as in the case of speaking rate, which seems to be affected by speaker's gender, education or occupation; see, e.g., [35, 36]). Besides duration, our Italian listeners also exploit the /s/ center of gravity, with higher values of mean frequency associated to higher gayness; differently, our German listeners exploit information concerning formant frequencies in some frontal vowels: the higher the F2 of /a/, /e:/, /ε/ and /I/, the more the speaker is perceived as gay. This same relation appears in a less coherent way in Italian (for a discussion of our results in relation to previous findings in English, see General Discussion). Interestingly, also /s/ center of gravity and formant frequency have been shown to be affected by social variation, suggesting that their features can be socially acquired and modified (on this issue, see, e.g., [37, 38])

The exploratory nature of our acoustic analyses does not allow us to conclude with certainty that longer duration and higher (second) formant frequencies are predictive of listeners’ judgments. However, we can state that relations between these measures emerged. It is worth noting that previous research has found differences between male and female speakers across languages that seem to resemble the current results. Indeed, males produce shorter vowel duration than females [39, 40]. Moreover, higher F2 values are reported in female compared to male speech (e.g., [4]). This suggests that listeners may, at least in part, derive their judgment of speaker’s SO from their knowledge of typical male vs. female speech styles. In line with this interpretation, the ratings of masculinity show that, at least for Italians, the perceived SO is related to perceived masculinity and there is some overlap in the speech cues listeners use to perform both the judgments. Surprisingly, this was not the case for Germans (for further discussion on this issue, see General Discussion).

To sum up, the results of the first 4 experiments showed that: a) the voice-based categorization of SO is fairly inaccurate; b) listeners tend to consistently categorize speakers’ SO on the basis of voice sound although this often does not reflect speakers’ self-identified SO; c) judgments are related to different types of acoustic cues, and although some cues are used in both languages (e.g., vowel duration), there is large variability across languages; d) at least for the languages under consideration, the categorization process is not *language-dependent* as it emerges in similar ways in distinct linguistic and cultural contexts; and e) it does not depend on the type of measure that is used. As a matter of fact, speakers were judged in a very similar way in Studies 1 and 2 (correlation between Italian samples of Experiment 1A and 2A: *r* (20) = .83, *p* <.001; correlation between German samples of Experiment 1B and 2B: *r* (12) = .67, *p* = .02).

## Experiment 3

### Cross-language categorization

The last experiment was designed to address the *language-specificity* issue. We tested whether Italian and German listeners show a similar pattern when categorizing the SO of speakers of their own vs. foreign language. Thus, besides replicating previous findings, Experiment 3 tested how listeners categorize foreign speakers and how they make their judgments in such cross-language categorization task.

### Method

#### Participants

One hundred and sixteen university students took part in this study. Eighty-six were recruited in Italy and 30 in Germany. Five participants reported to be gay or bisexual, two did not indicate their SO and two reported to be non-native speakers and thus were excluded from analyses. The final sample consisted of 107 heterosexual participants (79 Italians and 28 Germans; 35 males). Italian (*M*_*age*_ = 22.25, *SD* = 3.14) and German participants (*M*_*age*_ = 23.54, *SD* = 3.84) had similar age. The majority of them (84.1%) had no knowledge of the foreign language under consideration, whereas a minority (15.9%) reported to know some German/Italian but mainly at a very basic level. Analyses excluding participants with basic knowledge of the foreign language did not change the pattern of results.

#### Materials

Speakers were the same as in Experiments 1 and 2.

#### Procedure

Participants were instructed to listen to a set of voices and to judge the SO of each speaker. Stimuli were short sentences presented in two blocks: One block included sentences pronounced by 20 Italian speakers (10 self-identified as gay and 10 as heterosexual), and the other block included sentences by 12 German speakers (6 self-identified as gay and 6 as heterosexual). Each speaker pronounced the same sentence: “Il cane correva nel parco/Der Hund rannte durch den Park” [the dog was running in the park]. We chose this sentence because its length and structure is almost identical in the two languages. Participants heard first speakers of their native language and then those of the foreign language.

Using the same procedure as in Study 1B, Italian participants were randomly assigned to two conditions: they were either told initially that half of the 20 Italian and of the 12 German speakers were gay and half straight, or they received no information. Given the small sample of German participants, they were all assigned to the no-information condition.

After listening to each speaker, participants had to rate his SO on a Likert scale (from 1 = completely heterosexual to 6 = completely homosexual). Differently from Study 2, we used a 6-point scale, eliminating the ambiguous scale midpoint.

At the end of the experiment, participants reported their demographic information (gender, age, native language, and SO). In addition, we asked them to indicate whether, and to which degree, they knew the foreign language used in the experiment.

### Results

#### Preliminary analysis on Italian participants

We first tested whether the distribution information had any effect on Italian participants. Knowing the 50:50 distribution beforehand overall increased the gayness ratings from 3.13 in the no information condition to 3.40 in the 50:50 information condition, *F*(1,84) = 6.00, *p* < .016, *η*_*p*_^*2*^ = .07. Thus, knowing the distribution increased listeners’ subjective likelihood that a speaker may be gay. However, it did not increase the accuracy in any way as evidenced by the complete absence of an interaction between distribution information and speaker’s sexual orientation (*F* = 1.3, *p* > .25). We therefore compared Italian and German participants in all subsequent analyses without considering this factor any further.

#### Sexual orientation–Response accuracy for same vs. different language speakers

For exploratory purposes, we first simply looked at the percentage of speakers whose mean ratings fell on the heterosexual or on the gay pole of the scale (see [3], for a similar procedure). Overall, 81% of heterosexual speakers were collocated on the “correct side of the scale” (below 3.5), a percentage that reliably exceeded chance, binomial, *p* = .021, suggesting that the majority of listeners tended to judge them as heterosexual; importantly, this percentage was identical for judgments by same- and by different-language speakers. Gay speakers were collocated on the “correct side of the scale” (above 3.5) in only 62% of the cases when speakers and listeners spoke the same language, and in 69% of the cases when speakers and listeners spoke different languages, neither of which exceeded chance (see S5 Table for statistics for each speaker). Thus, overall hit rates were very similar for same- and other-language judgments. However, the more fine-grained analysis reported below suggests a more complex pattern.

#### Sexual orientation–Ratings for Italians and German speakers as a function of Listeners' nationality

A 2 (Listeners: Italian vs. German) x 2 (Speakers' language: Italian vs. German) x 2(Speakers' SO: gay vs. heterosexual) ANOVA with repeated measures on the last two variables was conducted on participants’ ratings (the higher the rating the more gay the speaker is perceived), considering the first factor as between-participants and the last two as within-participants variables. Overall, German listeners (*M* = 2.88, *SD =* .65) rated the speakers as more heterosexual than Italian listeners (*M* = 3.27, *SD =* .50), *F*(1,105) = 10.92, *p* = .001, *η*_*p*_^*2*^ = .16. Also, German speakers (*M* = 3.07, *SD =* .63) sounded more heterosexual than did Italian speakers (*M* = 3.27, *SD =* .56), *F*(1,105) = 37.72, *p* < .001, *η*_*p*_^*2*^ = .26. However, these main effects were modified by a significant interaction between Listeners’ and Speakers' language, *F*(1,105) = 4.78, *p* = .03, *η*_*p*_^*2*^ = .04. Although German listeners perceived speakers as more heterosexual than Italian listeners, this difference was more pronounced when judging German speakers (German listeners: *M* = 2.71, *SD =* .63, vs. Italian listeners: *M* = 3.19, *SD =* .57, *p <* .001) than when judging Italian speakers (German listeners: *M* = 3.04, *SD =* .70, vs. Italian listeners: *M* = 3.35, *SD =* .48, *p* < .01). Put differently, German listeners judged German speakers as particularly heterosexual.

Most important for the aims of our study is the fact that heterosexual speakers (*M* = 2.92, *SD =* .56) were judged as more heterosexual than gay speakers (*M* = 3.41, *SD =* .65), *F*(1,105) = 75.98, *p* < .001, *η*_*p*_^*2*^ = .42, suggesting that listeners overall tended to distinguish, to some extent, the two groups of speakers. However, this main effect was modified by an interaction with Listeners' language, *F*(1,105) = 39.27, *p* < .001, *η*_*p*_^*2*^ = .27, and by a three-way interaction between Listeners’ language, Speakers' language and Speakers' SO, *F*(1,105) = 7.46, *p* = .007, *η*_*p*_^*2*^ = .06. Pairwise comparisons corrected for multiple comparisons (Bonferroni’s correction) showed that Italian listeners rated heterosexual speakers as more heterosexual than gay speakers in both languages (Italian speakers: *F*(1,105) = 239.69, *p* < .001, *η*_*p*_^*2*^ = .69; German speakers: *F*(1,105) = 44.24, *p* < .001, *η*_*p*_^*2*^ = .30). German listeners tended to show the same pattern for German (heterosexuals: *M* = 2.60, *SD =* .63 and gays: *M* = 2.83, *SD =* .77; *F*(1,105) = 3.14, *p* = .07, *η*_*p*_^*2*^ = .03), but not for Italian speakers (heterosexuals: *M* = 3.05, *SD =* .70 and gays: *M* = 3.03, *SD =* .74; *F*(1,105) = .05, *p =* .82, *η*_*p*_^*2*^ = .00), suggesting a difficulty of German listeners to distinguish the SO of foreign speakers. Moreover, German listeners judged both the Italian heterosexual (*M* = 3.05, *SD =* .70) and gay speakers (*M* = 3.03, *SD =* .74) as less heterosexual/more gay than their German counterparts (heterosexuals: *M* = 2.60, *SD =* .63 and gays: *M* = 2.83, *SD =* .78; *F*s > 3.93, *p*s < .05). For Italian listeners this was true only for gay speakers (Italians: *M* = 3.73, *SD =* .50 and Germans: *M* = 3.45, *SD =* .69; *F*(1,105) = 21.57, *p* < .001, *η*_*p*_^*2*^ = .17), but not for heterosexual speakers of the two languages (*F*(1,105) = .17, *p* = .68, *η*_*p*_^*2*^ = .002). On one hand, this interaction shows that Italian listeners were better than German listeners in distinguishing SO of speakers in both languages. On the other hand, it suggests that Italians were better in recognizing SO of gay speakers of their own than those of a foreign language, whereas German listeners tended to judge Italian speakers as less heterosexual than German speakers regardless of their SO. Note however, that ratings referring to groups of speakers are overall low, suggesting that gay speakers were rarely identified correctly. Indeed, with the exception of Italian listeners judging Italian gay speakers (*t*(78) = —.68, *p* = .50), all means were below the midpoint of the scale (*t*s <- 3.38, *p*s < .002), suggesting an overall difficulty in rating speakers as gay.

#### Sexual orientation–Accuracy for Italians and German speakers as a function of Listeners' nationality

As in Studies 2A and 2B, we used within-participants correlations as dependent measure, representing the degree of agreement between the listener’s ratings and the speakers’ self-ratings of SO. Overall, there was a small but reliable agreement between self- and other-ratings (*M* = .18) that exceeded zero, one-sample *t*(115) = 11.67, *p* < .001. A 2 (Listeners: Italian vs. German) x 2 (Speakers' language: Italian vs. German) ANOVA with repeated measures on the second variable, using the within-participants correlations as dependent variable, revealed two main effects. Overall, the ratings of Italian listeners (*M* = .20, *SD* = .17) showed a larger agreement with the speakers’ self-ratings than the ratings of German listeners (*M* = .02, *SD* = .19), *F*(114) = 22.39, *p* < .001, *η*_*p*_^*2*^ = .17. Also, Italian speakers (*M* = .25, *SD* = .20) were recognized better than German speakers (*M* = .05, *SD* = .31), *F*(114) = 24.84, *p* < .001, *η*_*p*_^*2*^ = .18, independently of the listeners’ nationality. The interaction between the two variables was not significant (*p* = .26). Thus, although Italian listeners were more likely to be correct and Italian speakers were more likely to be recognized correctly, the absence of an interaction suggests that people were no better in guessing the SO for speakers of their own (vs. the other) language.

#### Perceived sexual orientation

**Fig 5:**
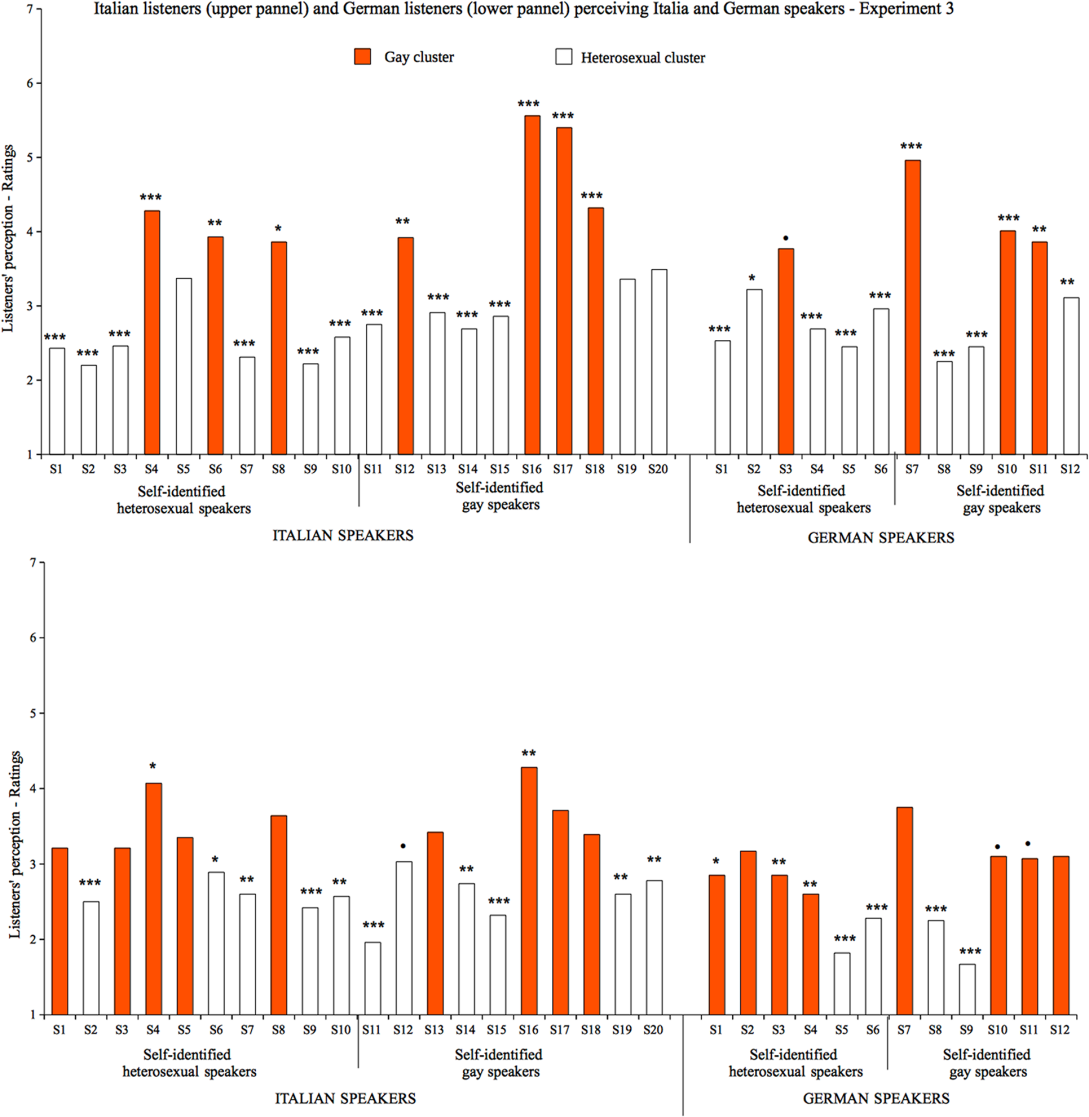
Italian (upper part) and German (lower part) listeners–in terms of ratings (Experiment 3)–of Italian and German speakers. Different colors indicate the two clusters according to listeners perception. Stars (and points) indicate those speakers significantly perceived either as heterosexual or gay (. < .1; * <.05; ** <.01; *** <.001). Higher values of the y-axis indicate perceived gayity, lower values perceived heterosexuality.

When one looks at the ratings for each speaker separately, it is evident that many speakers were incorrectly perceived with regard to their self-reported SO (Fig 5). For the Italian listeners, ratings of 1 German and 3 Italian speakers did not differ from the midpoint and the majority of self-identified Italian and German gay speakers were judged to be heterosexuals. This is evident in the k-means cluster analysis we performed on participants’ ratings for Italian and German listeners, separately. When considering Italian listeners, a two-cluster analysis for Italian speakers explained 70.6% of variability and yielded one cluster of heterosexual-sounding speakers that consisted of 13 speakers (mean value: 2.75) and another including 7 speakers (mean value: 4.47) perceived as sounding gay. The same analysis for German speakers accounted for 76.9% of data variability and showed one cluster of 8 heterosexual- sounding (mean value: 2.69) and another of 4 gay- sounding speakers (mean value: 4.12, see Fig 5).

The same analysis was performed on ratings of German listeners. A two-cluster analysis for German speakers explained 73.5% of variability and yielded one cluster of 4 speakers (mean value: 2.00) who clearly sounded heterosexual, and a second cluster of 8 speakers perceived still, but less strongly, as heterosexual (mean value: 3.05). The analysis for Italian speakers accounted for 70.2% of data variability and showed one cluster of 11 heterosexual-sounding (mean value: 2.56) and another cluster including 9 speakers that sounded less heterosexual (mean value: 3.57, see Fig 5).

#### Correlations between same and different language speakers

Our main interest was the degree of agreement between Italian and German listeners as they guessed the SO of Italian and German speakers. We therefore calculated, for each speaker, the mean SO rating provided by the Italian and the German sample. A high correlation (using speakers as unit of analysis) would suggest a high agreement regardless of whether the judgment is made for native or for foreign language speakers. Overall, there was considerable agreement between Italian and German listeners, *r*(32) = .75, *p* < .001. A separate look at Italian, *r*(20) = .74, *p* < .001, and German speakers, *r*(12) = .80, *p* = .002, suggests that “foreign” listeners largely agreed with the judgments of “native” listeners in both cases. Also, agreement between German and Italian listeners was of approximately equal strength when judging heterosexual, *r*(16) = .65, *p* = .006, and gay speakers, *r*(16) = .86, *p*< .001. Together, these data suggest that native-language and foreign-language judgments coincide to a remarkable degree. Thus, people tend to categorize the SO as well (or as poorly) in same-language as in different-language speakers.

Interestingly, although Italians and Germans agreed in their judgments to a large extent *r*(32) = .75, *p* < .001, correlations with the self-defined SO of the speakers was modest for Italian (*r*(32) = .41, *p* = .02) and even smaller for German listeners (*r*(32) = .17, *p* = .35).

### Discussion

Study 3 replicated and extended previous findings. The within-participants correlations suggest that there was a small but reliable degree of agreement between the listeners’ judgments and the speakers’ self-identified SO, such that speakers who rated themselves as less heterosexual were also perceived as less heterosexual. Nevertheless, in line with Studies 1 and 2, results show that listeners were often inaccurate in judging the SO of both, same and foreign language speakers. Accuracy was particularly low for self-identified gay speakers due to an overall tendency to judge speakers as heterosexuals. At the same time, categorization of speakers' SO seems not to happen by chance; rather listeners make a clear distinction on the basis of how voices sound. Importantly, this seems not to be a language-specific effect as shown by the general agreement between Italians and Germans' judgments.

The main question addressed in Study 3 was whether people are, to some degree, able to detect the SO in speakers whose language they do not comprehend. Two results argue in favor of this idea. First, our within-participants correlations showed no recognition advantage for own-language speakers. Overall, Italian listeners were more accurate and Italian speakers were identified more correctly, but this latter finding may in part be a methodological artifact as there were more Italian than german speakers. Hoewever, the absence of an interaction between speakers’ and listeners’ language suggests that own-language speakers were identified as good or a poor foreign-language speakers. Second, using speakers as a unit of analysis we found a very high agreement between the judgments of Italian and German listeners.

Thus, the most interesting finding emerging from Study 3 is that the judgments of native and foreign-language listeners converge to a surprising degree, yet neither seems to match the speakers’ self-defined SO very well. Thus, although accuracy is low, especially when guessing the SO of gays, the same speakers “sounded” heterosexual (or gay) to those who did and those who did not know the language.

Despite the similarity of result patterns across languages, a few interesting differences emerged. In our sample, German listeners seemed to be better at recognizing speakers' SO of same-language speakers, a result that is in line with Valentova and colleagues [21]. In contrast, Italian listeners' showed a very similar pattern in both languages.

Moreover, German listeners tended to perceive Italian speakers as always sounding less heterosexual/more gay than speakers of their own language, suggesting that some languages may sound less (or more) heterosexual than others to foreigners. Note that the extent to which a language is perceived as more (or less) heterosexual is a relative judgment since it probably refers to a direct comparison of the foreign language with the native language of the listeners. Therefore, Italian may not be a gay-sounding language in absolute terms, but it may appear such relative to German.

All in all, these results suggest that the process works similarly for speakers of different languages: Voice-based categorization of SO is not accurate but based on how listeners think a heterosexual and a gay voice should sound like. Moreover, the high agreement between “foreign” and “native” listeners' judgments, together with the low listeners' accuracy, suggest that the process is not *language-specific*.

## General Discussion

The present research aimed to shed light on the ongoing debate on the accuracy of voice-based categorization of SO across different languages. We first analyzed whether heterosexual listeners have an “ability” to correctly categorize the speakers' SO, and whether such voice-based categorization is consensual, such that different listeners concur in their judgments. Second, we wondered which acoustic cues speakers display and which cues listeners use to guess the speakers’ SO. Third, importantly, both issues (accuracy and acoustic cues) were investigated under a cross-language perspective to examine whether this voice-based categorization process is *language-dependent* or whether it operates in similar ways across languages and cultures. Finally, we tested whether the voice-based categorization of SO is *language-specific*, such that SO can only be detected by native-language speakers or also by those who are unfamiliar with the speaker’s language. These questions were addressed in five experiments in which listeners guessed the SO on the basis of short sentences that were pronounced either by self-identified heterosexual or gay speakers.

### Accuracy

Our experiments provide little evidence for the idea that people possess a reliable ability to detect SO from speakers’ voice. Across studies, participants showed rather low accuracy rates, especially when judging gay speakers, quite in contrast to some previous studies supporting the ability to correctly detect sexuality from voice [3, 5]. The comparably low hit rate in the current research may in part be due the fact that participants had to guess the SO of speakers on the basis of single sentences, whereas Gaudio [3] and Valentova and colleagues [5] used short paragraphs. However, Gaudio’s work seems to constitute an exception even within the body of English language studies, given that this study produced larger hit rates than any other study we are aware of. In fact, our findings seem largely in line with more recent studies conducted in English language, which challenge the idea of a reliable skill of detecting SO from voice alone (see [4, 6, 18]).

However, accuracy is not an all-or-none phenomenon and depends greatly on the test criterion used to establish whether a response is accurate or not. At a minimal level, any response reliably exceeding chance may be interpreted as a sign of accuracy. In this sense, our data provide supportive evidence since participants were more likely to judge heterosexual than gay speakers as heterosexual (all Experiments) and their ratings of SO correlated positively with the speakers’ self-ratings (see within-participant correlations, Exp. 2A, 2B and 3). Although gays were often misclassified, they were still perceived as *relatively* less heterosexual, suggesting some degree of accuracy. However, this interpretation becomes questionable when the magnitude of these effects is taken into account. In fact, the agreement between speakers’ self-ratings and listeners’ ratings of SO were generally of small magnitude (Exp. 2A, 2B), with only one medium size effect being observed in one of our studies (Exp. 3). Applying an even stricter criterion, one may look at our data in *absolute* terms, considering perceived SO as accurate only when a gay person is perceived as gay and a heterosexual person as heterosexual. In this sense, our data suggest a complete inability of listeners to identify the SO on the basis of voice alone, given that in all five experiments, judgments fall clearly and reliably short of meeting this criterion. Participants failed to correctly identify 1/3 of heterosexual and the large majority of gay speakers (Exp. 1A and 1B). Moreover, the same results emerged in all studies when we considered only exclusively heterosexual and gay speakers, that is when we conducted analyses excluding speakers who self-identified as 3 or 5 on the sexual orientation scale. This suggests that the extremity of self-identification as gay or heterosexual does not modify our results. In sum, listeners’ performance (slightly) exceeded chance when making relative distinctions in degree of heterosexuality, but judgments in absolute terms were close to chance (with heterosexuals being identified above, but gay speakers below chance.

Across all studies, we found a very consistent difference in the capacity to detect the SO of heterosexual vs. gay speakers. Whereas the correct recognition of heterosexuals exceeded chance (66% across all studies), only a very small minority of gay speakers was correctly identified as such (19% across all studies). Thus, participants tended to incorrectly perceive gay males as heterosexual and, somewhat surprisingly, this was even true when listeners had been informed that half of the speakers were heterosexual and half gay (Exp. 1B and 3A).

Likelihood estimates should logically be influenced by prior assumptions of base-rates (see [41]). For instance, participants may assume that voice samples are taken randomly from the population and that, as a consequence, heterosexual voices should greatly outnumber gay voices. This may explain why “heterosexual” becomes the default option when guessing the speakers’ SO. To our knowledge, this is the first research in which prior knowledge of the distribution of gay and heterosexual speakers was manipulated experimentally (Study 1B in German and Study 3 in Italian). In one of the two studies (Study 3) knowing the distribution did, indeed, increase the listeners’ subjective likelihood that a speaker may be gay. However, in neither study did this knowledge affect the accuracy in any way. Thus, although additional research is needed before making definite claims, our findings suggest that prior information about the distribution of gay and heterosexual people in a population may create a response bias, but may not necessarily affect accuracy.

The mouse tracker findings in our first two studies provide converging evidence for the idea that *heterosexuality* constitutes the dominant response. Whenever people identified a speaker as gay, they tended to provide their answers with greater uncertainty (resulting in a larger curve), as if they were attracted towards the *heterosexual* pole. Together, both outcome (accuracy rates) and process measures (mouse tracking) suggest that most male voices (even those of gays) sounded heterosexual to the ears of our heterosexual listeners and that they were reluctant to identify speakers as gay. On a methodological note, accuracy in our studies was low regardless of the specific measure used. In two studies (Exp. 1A and 1B) participants were asked to categorize speakers through dichotomous choices and under time pressure, whereas in 3 studies (Exp. 2A, 2B and 3) they expressed their judgments on a Likert scale and with no response time limit. Despite these methodological differences the studies yielded very similar result patterns, all showing consistently low accuracy rates.

Despite the modest accuracy rates, we observed a considerable consensus among listeners. A subgroup of gay speakers was reliably identified as gay across studies and across languages (in particular, speakers 16 and 17 of the Italian sample and, with only one exception, speaker 7 of the German sample). This suggests that the modest mean differences observed between the two groups of speakers are due to a small subgroup of clearly gay-sounding speakers. Apparently, there is a great variability in speech styles within both groups and only a small percentage of gay speakers are clearly identifiable as such. Whether these speakers are deliberately trying to communicate their SO remains an interesting question to investigate in the future.

By the same token, some gay speakers were consistently misidentified as heterosexual (speakers 11, 14 and 15 of the Italian sample and speakers 8 and 9 of the German sample) and some heterosexual speakers tended to be misidentified as gay (speaker 4 in the Italian sample and, less coherently, speakers 2 and 3 in the German sample). Thus, although participants tended to provide inaccurate guesses, they did not answer by chance, as shown by the fact that many speakers were consistently categorized either as heterosexual or gay regardless of their actual SO. In all studies, there was a surprising agreement between listeners suggesting that there was something about the speakers’ voice that made people draw conclusions about their (presumed) SO. Some speakers simply sounded more gay or heterosexual, regardless of their self-defined SO. But what cues let people to assume that a speaker was gay or heterosexual?

### Acoustic cues used to express and to identify sexual orientation

Given the high agreement among listeners, it is likely that their judgments were related to specific acoustic features, meaning that the way of speaking may have influenced perceived SO. Under a comparative perspective, it is important to note that there is no consistency between the acoustic cues used by listeners of the different languages under investigation here (i.e., Italian and German) or those previously examined in English. Thus, for example, while some properties of /s/ have been reported to be relevant for British or American English speakers (e.g., [5]) and Italians (Experiment 1A and 2A), they are not for Germans; similarly, German listeners, as well as English listeners [4], tend to exploit the vowels formant frequencies more than Italians did.

An interesting acoustic feature that seems to play some role in both Italian and German is sound duration: listeners tended to categorize as gay those speakers who produced longer sounds. Note that sound duration has large within-speaker variability and may be affected by variables such as the speaker’s intention and other social variables (e.g., education, occupation; see for instance, [36]). The same is true for /s/ center of gravity, which can vary as a function of the speaker’s social identity (see, [42, 43]). Therefore, if the cues used to identify SO are features that are subject to social variation rather than anatomically determined, this may also explain why different acoustic cues are used in different cultures and languages to express and to interpret SO. Thus, the stereotype of a heterosexual/gay voice may vary across cultures and languages. This interpretation is in line with other studies that have shown that the same characteristics can be communicated through different means in different languages. For instance, D’Errico and colleagues [40] have shown that *charisma* is communicated through different vocal cues in different language communities.

It should be noted that the cues used to guess the SO of speakers show little overlap with the cues that objectively distinguish (self-identified) gay from heterosexual speakers. For the German sample, we were unable to identify acoustic cues that were used to express (speakers) and to guess homosexuality (listeners). For Italians, vowel duration seems to be the best candidate for explaining why Italian listeners judged gay speakers as less heterosexual than heterosexual speakers, given that this cue distinguished self-identified gay speakers and was also used by listeners to guess the speakers’ SO. This may also explain why Italian speakers were recognized more correctly than German speakers by both Italian and German listeners in Study 3.

### Gay voice and masculinity

In our research, we also found some relation between the perception of SO and masculinity. Specifically, among Italian listeners, perception of sexual orientation seems to go hand in hand with perception of masculinity from voice. This is supported by the fact that some acoustic cues (center of gravity of /s/) are positively correlated with both listeners’ ratings of sexual orientation and masculinity. In the German sample no relation was found between perceived sexual orientation and masculinity. However, German listeners rated as more gay-sounding those speakers who had acoustic features typically related to female speech (e.g., [44]). Under this perspective, speakers may be more likely to be perceived as gay when they display a speech style that contains more typically female features. If we consider the speech features as placed on a male-to-female continuum, the closer a speech feature is placed to the female end of the continuum, the more the male voice that produces it will sound gay.

The proximity of the acoustic properties of gay speech to those of female speech had already been proposed by Smyth and Rogers [36], who highlighted that, in English, gay-sounding and female voices share some acoustic features, such as the peak spectral frequency of /s/ and (some) vowels duration. Note that the acoustic features shared by gay-sounding and female speech can all vary according to social dimensions (e.g., the frequency properties of /s/ may vary according to social class; [38]). This parallels our findings and suggests that the gay-sounding voice has a social, not a physical, origin. If so, where does the gay-sounding voice come from? Smyth and Rogers [37] suggest that the gay-sounding voice is modeled on the female speech and tends to develop in gender nonconforming boys, that is in boys who are strongly psychosocially linked to females and use their speech as a model. In this way, gender nonconforming boys will acquire phonetic features typical of female speech, with the consequence that their voice will sound gay to the listeners' ear (for a similar proposal, see also [45]).

The theory proposed by Smyth and Rogers is indeed intriguing and our data offer indirect support for it, especially for our Italian sample, in which the perception of gayness was related to that of effeminateness, both in terms of listeners’ ratings and acoustic features related to their judgments. However, the fact that we found no direct relation between the perception of gayness and effeminateness in German suggests some caution. One possibility is that the lack of relation is due to the small sample of speakers. Alternatively, given that the acoustic features that make a voice sound gay are subject to social and cultural variations, it may also be the case that the gender nonconformity and the tendency to model own speech after that of female models varies as a function of the social context and culture, with a consequent effect on the relation between perceived gayness and effeminateness. The issue is particularly interesting and deserves further investigation by future psycholinguistic and social research.

### Language-dependency

Closely related to the above questions, is the third issue we addressed, namely that of *language-dependency* [14, 39]. Previous studies had focused only on English listeners and speakers; in the present study we added results from two different languages–Italian and German–which are very different from one another at multiple levels, including their phonological system and its phonetic realization (e.g., different number of vowels, different tendency to phonological reduction). Moreover, Italian and German are spoken in two countries with different laws, cultures, and attitudes toward homosexualityity. Despite such differences, our findings provide evidence that the voice-based categorization of speakers’ SO works, in some ways, similarly in the two languages (and countries) investigated here.

In both languages we found that (a) judgments of SO are highly inaccurate, especially for gay speakers, (b) that listeners are reluctant to identify speakers as gay (although this reluctance was more pronounced among German listeners), and (c) that listeners consistently classified certain speakers as gay or heterosexual, largely independent of their actual SO and probably driven by listeners’ expectations of what male heterosexual voices sound like. This result helps to interpret previous findings, obtained in English language communities, in a broader perspective that looks at the ability to detect SO from voice as an inaccurate tool driven by listeners’ expectations. Thus, the general process guiding the perception of SO on the basis of voice seems to be similar across the three languages studied so far (English, Italian, German), suggesting that listeners use comparable, language-independent strategies. These findings seem to differ from those of Valentova and colleagues [5] as they found an overall good accuracy. It is worth noting that even in their case, means were low or around the midpoint of the scale, meaning a tendency to consider heterosexuality as the default. Moreover, their results may be specific for the types of listeners considered in their study, namely heterosexual women and gay men, leaving out heterosexual men. In our case, we only considered heterosexuals and we did not find gender differences across five studies. We investigated acoustic cues and found that specific acoustic cues are used to infer SO in each linguistic context. It is conceivable that stereotypic expectations of how male heterosexual vs. gay voices sound are, to some degree, culture-specific, but that the general process of expectancy-driven judgments is analogous.

### Language specificity

The last issue addressed in our studies refers to *language-specificity* that is the question whether listeners are able to recognize the SO of an individual who speaks a different language. Our results seem to show that the process is not language-specific, but that it works similarly with speakers of distinct languages. Differently from previous research by Valentova et al. [21], our cross-cultural experiments showed that listeners are incorrect in judging SO of both own and other language speakers. The different pattern of results may be easily explained by the fact that the categorization was based solely on voice in our studies, whereas in Valentova's study [21] participants had both vocal and visual information available when judging the targets’ SO. Thus, the availability of a more complex and rich signal system could indeed facilitate the recognition of people SO, which is an ambiguous feature that is difficult to detect. Nonetheless, both Italian and German listeners in our studies made a distinction between groups of speakers who were perceived as sounding gay or heterosexual, and this was true for both same- and other-language speakers. In fact, there was a surprisingly high agreement (*r* = .75) between the judgments of Italian and German listeners, although these judgments rarely coincided with the self-identified SO of the speakers. Although we have no data directly speaking to the question of what cues listeners use when judging the SO of foreign speakers, we may speculate that the process occurs on the basis of the cues of one’s own language: When rating foreign speakers, listeners might rely on those cues that they associate to gayness in their native language. Thus, Germans may rate Italian speakers based on duration and F2 measures, whereas Italians may base their ratings of German speakers on durational measures and /s/ center of gravity. In our data, durational measures were important for listeners of both languages and might, at least in part, explain the agreement in the judgments of listeners of the languages.

However, we cannot exclude that language characteristics other than the acoustic features investigated here might affect the listeners' judgment. In fact, an interesting, unpredicted finding was that Italian speakers sounded, on average, more gay than German speakers to both Italian and German listeners. It is conceivable that some languages such as Italian and French sound more melodic or “feminine”, whereas others such as German may sound more masculine (for empirical support see [44, 46, 47]), which in turn may make their speakers appear more or less gay-sounding. However, the perception of languages may not be absolute and universal. Italian may be a gay or feminine language for German, but not for listeners of other languages. If supported by future research across larger samples of languages, this may be a socially relevant case of *sound symbolism*, according to which sounds reveal meaning and promote images, a principle that has also been deployed in the creation of brand labels [48].

### Limits and open questions

Although this is, to our knowledge, the first study investigating voice-based categorization of SO (independent of other cues such as facial features) under a cross-cultural perspective, it unfortunately shares a number of limits with prior research. Most importantly, it involves a limited and unrepresentative sample of speakers (in all 32 speakers); this methodological constraint poses limits to the generalizability of our findings to the general population of gay and heterosexual speakers. The same can be said about the selection of sentences that are clearly unrepresentative of naturally occurring discourse. To better understand and interpret these findings, future studies should replicate these results with larger and more representative samples of speakers and stimuli.

An additional limit of the present studies is that they focus exclusively on heterosexual listeners. It remains to be seen whether gay listeners develop better recognition skills either due to greater exposure to and experience with gays or due to greater attention and/or motivation. Thus, research is needed that includes gay populations not only among speakers but also among listeners. Indeed, although research suggests that gay men and lesbian women are more accurate in judging SO than their heterosexual counterparts on the basis of short observation of non-verbal behavior and visual stimuli [23], no studies have investigated this issue with regards to voices.

Given that this is the first attempt to investigate this voice-based categorization process under a cross-linguistic perspective, not be surprising that many questions remain unanswered. First of all, it remains to be seen whether the relatively similar pattern observed in two languages (German and Italian) will be found across other Indo-European languages (e.g., Spanish, French, Portuguese, etc.) and will also hold when investigating languages of different language families such as Chinese, Korean or Swahili.

Second, it remains to be investigated to what extent gay-sounding voices contain features of female speech and whether this is due to the fact that gay-sounding voices belong to people that in their childhood were strongly psychosocially linked to females and used female speech as a model. This issue should be addressed also in terms of cross-cultural comparison in order to identify the role of social and cultural variation on gay voice to female voice association.

Third, it remains to be investigated why listeners rely on acoustic cues that are only in small parts veridical and why people from different languages rely on different cues. On one side, different languages offer different affordances (e.g. not all vowels or consonants are present in all languages), on the other side people may learn to associate different ways of speaking with SO in culture-specific ways. For instance, films and other media appear to associate gay protagonists, frequently played by heterosexual actors, with specific speech styles that may differ from language to language. If stereotypic expectancies of listeners derive, at least in part, from the way gay speech is portrayed in the media, rather than from exposure to actual gay people, this would explain why people from different cultures develop distinct expectations.

Forth, it remains to be investigated whether the acoustic cues used to express or to infer SO are under the voluntary control of speakers or whether they are anatomically determined. If cues are at least in part under the speakers’ control, as our duration measures suggest, then one would expect considerable cross-cultural variation in the public display of such speech styles by gay speakers, given that countries differ greatly in their acceptance of homosexuality and in the rights granted to gays. A similar argument can be made for cross-situational and cross-time variations. Revealing one’s SO (be it heterosexual or gay) is clearly functional in many situations, but since, for gays, it still implies some risk of discrimination one would expect some degree of variability (e.g. in work settings vs. at bars or before vs. after coming out). The degree to which revealing speech styles change over time and across contexts remains an interesting question for future investigation.

### Conclusion

The present research shows that the way we categorize individuals does not always correspond to reality. Voice can be informative about the speakers, without necessarily being accurate. Especially in the case of a feature such as SO that can be easily hidden, people should be aware that their perception may be misleading and that their (correct or incorrect) inferences may change their interpersonal behaviors. Implicit inferences from voice can easily lead to stereotypes and sexual discrimination. Imagine an everyday situation where people listen to the voice of a male individual on the phone. Categorizing him as gay or perceiving him as low in masculinity on the basis of his voice might lead people to believe that he is creative, but fragile and unfit for leadership roles [49]. Therefore, the simple exposure to a voice is sufficient not only to drive the speaker's categorization, but also to make stereotypical inferences about his/her life.

Together, the present research suggests that the categorization of SO from voice functions on the bases of audience expectations rather than as an accurate detector of speakers' SO. Across languages and countries, voice has emerged as a highly informative cue that people use to consistently (and often erroneously) categorize individuals.

## Appendix

Sentences (in English translation) used in Experiments 1B and 2B.

[*Common name]* bought a copy of the newspaper.

Today, [*common name]* has received a postcard.

The English course begins on Monday.

[*Common name]* has bought a new cd.

The cat always plays with the ball of yarn on the couch.

[*Common name]* always drinks tea when it's cold.

[*Common name]* frequently shops online.

This car has done 350 km.

Yesterday, the chicken did two eggs.

Yesterday, the pastor has shorn his sheep.

[*Common name]* parked near the historic center.

[*Common name]* very often takes her bike.

The dog was running in the park.

The movie theatre has just changed the show times.

[*Common name]* took a plane only once.

[*Common name]* often uses internet with the cell phone.

[*Common name]* goes every week to the mall.

Yesterday, [*common name]* took the car to the mechanic.

[*Common name]* has forgotten her running shoes in the car.

[*Common name]* has assembled the book shelves in an hour.

Sentences (in Italian) used in Experiments 1A and 2A.

Marco ha comprato una copia del giornale.

Oggi Luca ha ricevuto una cartolina.

Il corso di inglese inizia lunedì.

Maria ha comprato un cd nuovo.

Il gatto gioca sempre con il gomitolo sulla poltrona.

Franca beve sempre il tè quando fa freddo.

Mattia fa spesa online di frequente.

Questa macchina ha fatto 350 chilometri.

Ieri la gallina ha fatto due uova.

Ieri il pastore ha tosato le sue pecore.

Marco ha parcheggiato vicino al centro storico.

Francesca va molto spesso in bici.

Il cane correva nel parco.

Il cinema ha appena cambiato gli orari degli spettacoli.

Piero ha preso l’aereo solo una volta.

Luca naviga spesso su internet col cellulare.

Valeria va al centro commerciale tutte le settimane.

Ieri Luca ha portato la macchina dal meccanico.

Martina ha dimenticato le scarpe da corsa in macchina.

Marco ha montato la libreria in un'ora.

Sentences (in German) used in Experiments 1B and 2B.

Andreas hat eine Zeitung gekauft.

Heute hat Friederike eine Postkarte geschickt bekommen.

Der Englischkurs beginnt am Montag.

Maria hat eine neue CD gekauft.

Die Katze spielt immer mit einem Wollknäuel auf dem Sofa.

Bastian trinkt immer Tee, wenn es kalt ist.

Matthias kauft oft im Internet ein.

Dieses Auto ist 350 Kilometer gefahren.

Gestern hat das Huhn zwei Eier gelegt.

Gestern hat der Schäfer seine Schafe geschoren.

Bruno hat in der Nähe des Stadtzentrums geparkt.

Paula fährt oft mit dem Fahrrad.

## Supporting Information

S1 Table Experiment 1A & 1B: Statistics for each Speaker. Legend:. .1; * < .05; ** < .01; *** < .001; ns: non significant; cluster: 1 = heterosexual; 2 = homosexual
(DOC)

S2 Table Familiarity with and attitudes toward gay men. (DOCX)

S3 Table Experiment 2A & 2B: Statistics for each Speaker. Legend:. .1; * < .05; ** < .01; *** < .001; ns: not significant; Cluster: 1 = heterosexual; 2 = homosexual
(DOC)

S4 Table Correlation between acoustic cues and listeners judgments. (DOC)

S5 Table Statistics for each Speaker. Legend:. .1; * < .05; ** < .01; *** < .001; ns: not significant; Cluster: 1 = heterosexual; 2 = homosexual
(DOC)
