# Supplementary material for: The Sound of Voice: Voice-Based Categorization of Speakers’ Sexual Orientation within and across Languages
Source: PLoS One. 2015 Jul 1;10(7):e0128882. doi: 10.1371/journal.pone.0128882 (PMC4488841; doi:10.1371/journal.pone.0128882)
Supplement: S2 Table — (DOCX) [file pone.0128882.s002.docx]

SM 2

Familiarity with and attitudes toward gay men

Familiarity with and attitudes toward gay men have been found to be related to categorization of sexual orientation (mostly face-based categorization; [1, 2]). In some of our experiments, we measured the number of gay contacts/friends and the closeness to the gay person participants knew best (Inclusion of Other in Self (IOS); [3]), Attitudes Toward Gay men (ATG; [4]), and Traditional Beliefs in Gender Identity (TBGI; [5]). Below we report correlations between these measures and speakers’ ratings of perceived sexual orientation and perceived masculinity across studies. Please note that the same measures were not consistently measured in all experiments. Across experiments, our analyses suggest no relation between the variables under consideration and listeners’ judgments.

|  |  | *Gay contacts* | *IOS* | *ATG* | *TBGI* |
| --- | --- | --- | --- | --- | --- |
| *Perceived sexual orientation* | Study 2A | *r* = .009, *p* > .9 | *r* = .110, *p* > .5 | *r* = -.148, *p* > .4 | *r* = -.098, *p* > .6 |
|  | Study 2B | --- | ---- | *r* = -.106, *p* > .5 | *r* = -.156 *p* > .4 |
|  | Study 3 | *r* = .140, *p* > .2 | --- | ---- | ----- |
| *Perceived masculinity* | Study 2A | *r* = -.163, *p* > .3 | *r* = .213, *p* > .2 | *r* = .221, *p* > .2 | *r* = .205, *p* > .2 |
|  | Study 2B | *---* | --- | *r* = -.165, *p* > .3 | *r* = .042, *p* > .8 |

References

1. Brambilla M, Riva P, Rule NO (2013). Familiarity increases the accuracy of categorizing male sexual orientation. Pers Indiv Differ, 55: 193-195.

2. Rule NO, Tskhay KO, Brambilla M, Riva P, Andrzejewski SA, Krendl AC (2015). The relationship between anti-gay prejudice and the categorization of sexual orientation. Pers Indiv Differ, 77: 74-80.

3. Aron A, Aron EN, Smollan D (1992). Inclusion of Other in the Self Scale and the structure of interpersonal closeness. J Pers Soc Psychol, 63: 596-612.

4. Herek GM (1998). Attitudes toward lesbians and gay men scale. *Handbook of sexuality-related measures*, 392-394.

5. Dasgupta N, Rivera LM (2006). From automatic antigay prejudice to behavior: the moderating role of conscious beliefs about gender and behavioral control. J Pers Soc Psychol, 91: 268-280.
