# Supplementary material for: The Sound of Voice: Voice-Based Categorization of Speakers’ Sexual Orientation within and across Languages
Source: PLoS One. 2015 Jul 1;10(7):e0128882. doi: 10.1371/journal.pone.0128882 (PMC4488841; doi:10.1371/journal.pone.0128882)
Supplement: S3 Table — Legend:. .1; * < .05; ** < .01; *** < .001; ns: not significant; Cluster: 1 = heterosexual; 2 = homosexual (DOC) [file pone.0128882.s003.doc]

SM2

Experiment 2A (Italians). Mean ratings, analyses and cluster values for each speaker.

| Speaker | Self-reported SO | Mean rating value | t-test | p value | Cluster |
| --- | --- | --- | --- | --- | --- |
| Speaker 01 | heterosexual | 2.28 (1.46) | - 6.35 | *** | 1 |
| Speaker 02 | heterosexual | 2.34 (1.26) | -6.91 | *** | 1 |
| Speaker 03 | heterosexual | 1.69 (.85) | -14.65 | *** | 1 |
| Speaker 04 | heterosexual | 3.97 (1.57) | - .12 | Ns | 2 |
| Speaker 05 | heterosexual | 2.72 (1.44) | - 4.78 | *** | 1 |
| Speaker 06 | heterosexual | 2.52 (1.66) | - 4.81 | *** | 1 |
| Speaker 07 | heterosexual | 2.28 (1.13) | - 8.21 | *** | 1 |
| Speaker 08 | heterosexual | 2.62 (1.50) | - 4 .96 | *** | 1 |
| Speaker 09 | heterosexual | 2.34 (1.47) | - 6.06 | *** | 1 |
| Speaker 10 | heterosexual | 2.28 (1.10) | - 8.45 | *** | 1 |
| Speaker 11 | homosexual | 2.72 (1.22) | -5.62 | *** | 1 |
| Speaker 12 | homosexual | 3.10 (1.26) | -3.82 | ** | 1 |
| Speaker 13 | homosexual | 1.52 (.69) | -19.44 | *** | 1 |
| Speaker 14 | homosexual | 2.24 (1.33) | -7.14 | *** | 1 |
| Speaker 15 | homosexual | 1.66 (.81) | -15.51 | *** | 1 |
| Speaker 16 | homosexual | 5.72 (1.39) | 6.70 | *** | 2 |
| Speaker 17 | homosexual | 4.90 (1.61) | 2.99 | ** | 2 |
| Speaker 18 | homosexual | 3.28 (1.85) | -2.11 | * | 1 |
| Speaker 19 | homosexual | 3.45 (1.74) | -1.70 | Ns | 2 |
| Speaker 20 | homosexual | 3.79 (1.70) | -.66 | Ns | 2 |

. < .1; * < .05; ** < .01; *** < .001; ns: not significant; Cluster: 1 = heterosexual; 2 = homosexual

Table 4. Experiment 2B (Germans). Mean ratings, analyses and cluster values for each speaker.

| Speaker | Self-reported SO | Mean rating value  (standar deviation) | t-test | p value | Cluster |
| --- | --- | --- | --- | --- | --- |
| Speaker 002 | heterosexual | 1.86 (1.33) | - 9.62 | p < .001 | 1 |
| Speaker 003 | heterosexual | 4.33 (1.93) | 1.04 | p = .31 | 2 |
| Speaker 007 | heterosexual | 2.33 (1.33) | - 7.51 | p < .001 | 1 |
| Speaker 009 | heterosexual | 2.97 (1.71) | - 3.59 | p = .001 | 1 |
| Speaker 011 | heterosexual | 2.42 (1.56) | - 6.10 | p < .001 | 1 |
| Speaker 013 | heterosexual | 2.36 (1.22) | - 8.04 | p < .001 | 1 |
| Speaker 006 | homosexual | 2.83 (1.75) | - 4.00 | p < .001 | 1 |
| Speaker 015 | homosexual | 2.83 (1.46) | - 4.78 | p < .001 | 1 |
| Speaker 027 | homosexual | 2.39 (1.57) | - 6.15 | p < .001 | 1 |
| Speaker 029 | homosexual | 3.86 (1.87) | -.45 | p = .66 | 2 |
| Speaker 030 | homosexual | 3.39 (1.74) | - 2.10 | p = .04 | 2 |
| Speaker 031 | homosexual | 3.64 (1.85) | -1.17 | p = .25 | 2 |

Cluster: 1 = heterosexual; 2 = homosexual
