# Supplementary material for: The Sound of Voice: Voice-Based Categorization of Speakers’ Sexual Orientation within and across Languages
Source: PLoS One. 2015 Jul 1;10(7):e0128882. doi: 10.1371/journal.pone.0128882 (PMC4488841; doi:10.1371/journal.pone.0128882)
Supplement: S4 Table — (DOC) [file pone.0128882.s004.doc]

SM3

Correlation between acoustic cues and listeners judgments of speakers' sexual orientation in Experiment 1A and 2A.

|  | Experiment 1A | | Experiment 2A | |
| --- | --- | --- | --- | --- |
|  | Correlation with Homo-index | *p* value | Correlation with speakers' rate | *p* value |
| Vowel F0 |  |  |  |  |
| /a/ | ρ = .24 | >.3 | ρ = .01 | >.9 |
| /e/ | ρ = .21. | >.3 | ρ = -.08. | >.7 |
| /i/ | ρ = .33 | >.1 | ρ = .20 | >.3 |
| /o/ | ρ = -.332 | >.1 | ρ = -.12 | >.9 |
| /u/ | ρ = .26 | >.2 | ρ = -.01 | >.9 |
| Vowel F1 |  |  |  |  |
| /a/ | ρ = .19 | >.4 | ρ = .20 | >.4 |
| /e/ | ρ = -.27 | >.2 | ρ = -.18 | >.4 |
| /i/ | ρ = .18 | >.4 | ρ = .48 | =.03 |
| /o/ | ρ = .11 | >.6 | ρ = .25 | >.2 |
| /u/ | ρ = -.20 | >.4 | ρ = -.23 | >.3 |
| Vowel F2 |  |  |  |  |
| /a/ | ρ = .55 | =.01 | ρ = .39 | =.09 |
| /e/ | ρ = .67 | <.001 | ρ = .31 | >.1 |
| /i/ | ρ = .22 | >.3 | ρ = .22 | >.3 |
| /o/ | ρ = .29 | >.2 | ρ = .40 | =.08 |
| /u/ | ρ = -.03 | >.9 | ρ = -.13 | >.5 |
| Vowel Duration |  |  |  |  |
| /a/ | ρ = .47 | =.03 | ρ = .40 | =.08 |
| /e/ | ρ = .44 | =.05 | ρ = .32 | >.1 |
| /i/ | ρ = .53 | =.02 | ρ = .50 | =.03 |
| /o/ | ρ = .50 | =.02 | ρ = .47 | =.03 |
| /u/ | ρ = .44 | =.05 | ρ = .26 | >.2 |
| Mean vowel duration | ρ = .54 | =.01 | ρ = .46 | =.04 |
|  |  |  |  |  |
| /s/ measures |  |  |  |  |
| Center of gravity | ρ = .57 | =.01 | ρ = .52 | =.02 |
| skewness | ρ = -.45 | =.06 | ρ = -.39 | =.09 |
| kurtosis | ρ = -.09 | >.7 | ρ = -.05 | >.8 |
| duration | ρ = .20 | >.3 | ρ = -.04 | >.8 |
|  |  |  |  |  |
| Speaking rate | ρ = -.72 | <.001 | ρ = -.53 | =.02 |

Correlation between acoustic cues and listeners judgments of speakers' masculinity (Experiment 2A).

|  | Correlation with sexual orientation rate | *p* value |
| --- | --- | --- |
| Vowel F0 |  |  |
| /a/ | r = .04 | >.8 |
| /e/ | r = .12 | >.6 |
| /i/ | r = .15 | >.5 |
| /o/ | r = .28 | >.2 |
| /u/ | r = .09 | >.7 |
| Vowel F1 |  |  |
| /a/ | r = .09 | >.6 |
| /e/ | r = .38 | >.1 |
| /i/ | r = -.33 | >.1 |
| /o/ | r = .18 | >.4 |
| /u/ | r = .29 | >.2 |
| Vowel F2 |  |  |
| /a/ | r = .15 | >.5 |
| /e/ | r = -.06 | >.8 |
| /i/ | r = -.35 | >.1 |
| /o/ | r = -.29 | >.2 |
| /u/ | r = .07 | >.7 |
| Vowel Duration |  |  |
| /a/ | r = .11 | >.6 |
| /e/ | r = -.12 | >.6 |
| /i/ | r = -.23 | >.3 |
| /o/ | r = -.20 | >.3 |
| /u/ | r = .11 | >.6 |
| Mean vowel duration | r = -09. | >.7 |
|  |  |  |
| /s/ measures |  |  |
| Center of gravity | r = .46 | =.04 |
| skewness | r = .46 | =.04 |
| kurtosis | r = .42 | =.07 |
| duration | r = .19 | >.4 |
|  |  |  |
| Speaking rate | r = . |  |

Correlation between acoustic cues and speakers self-identification for the Italian sample.

|  | Correlation with sexual orientation rate | *p* value |
| --- | --- | --- |
| Vowel F0 |  |  |
| /a/ | ρ = -.22 | >.3 |
| /e/ | Ρ = -.18 | >.4 |
| /i/ | ρ = .01 | >.9 |
| /o/ | ρ = -.21 | >.3 |
| /u/ | ρ = -.25 | >.2 |
| Vowel F1 |  |  |
| /a/ | ρ = .13 | >.6 |
| /e/ | ρ = .17 | >.4 |
| /i/ | ρ = .46 | =.04 |
| /o/ | ρ = .37 | >.11 |
| /u/ | ρ = .51 | =.02 |
| Vowel F2 |  |  |
| /a/ | ρ = .40 | =.08 |
| /e/ | ρ = -.29 | >.2 |
| /i/ | ρ = -.06 | >.7 |
| /o/ | ρ = .32 | >.1 |
| /u/ | ρ = .14 | >.5 |
| Vowel Duration |  |  |
| /a/ | ρ = .45 | =.05 |
| /e/ | ρ = .40 | =.08 |
| /i/ | ρ = .38 | >.1 |
| /o/ | ρ = .59 | =.01 |
| /u/ | ρ = .02 | >.9 |
| Mean vowel duration | ρ = .46 | =.04 |
|  |  |  |
| /s/ measures |  |  |
| Center of gravity | ρ = .25 | >.2 |
| skewness | ρ = -.26 | >.2 |
| kurtosis | ρ = .03 | >.8 |
| duration | ρ = .16 | >.5 |
|  |  |  |
| Speaking rate | ρ = .22 | >.3 |

Correlation between acoustic cues and listeners judgments in Experiment 1B and 2B.

|  | Experiment 1B | | Experiment 2B | |
| --- | --- | --- | --- | --- |
|  | Correlation with Homo-index | *p* value | Correlation with speakers' rate | *p* value |
| Vowel F0 |  |  |  |  |
| /a/ | r = .10 | >.7 | r = .14 | >.6 |
| /a:/ | r = .15 | >.6 | r = .15 | >.6 |
| /e:/ | r = .17 | >.6 | r = .25 | >.4 |
| /ɛ/ | r = -.01 | >.9 | r = -.13 | >.6 |
| /ɪ/ | r = -.15 | >.6 | r = -.25 | >.4 |
| /i/ | r = -.12 | >.7 | r = -.04 | >.8 |
| /i:/ | r = .28 | >.3 | r = .30 | >.3 |
| /o/ | r = .07 | >.8 | r = .01 | >.9 |
| /o:/ | ρ= .27 | >.3 | ρ = -.13 | >.6 |
| /u:/ | r = .28 | >.3 | r = .24 | >.4 |
| Vowel F1 |  |  |  |  |
| /a/ | r = .44 | >.1 | r = .57 | =.06 |
| /a:/ | r = .45 | >.1 | r = .58 | =.06 |
| /e:/ | r = .01 | >.9 | r = -.28 | >.3 |
| /ɛ/ | r = .32 | >.3 | r = .32 | >.3 |
| /ɪ/ | r = .27 | >.4 | r = .01 | >.9 |
| /i/ | r = .30 | >.3 | r = -.18 | >.5 |
| /i:/ | r = .01 | >.9 | r = -.16 | >.6 |
| /o/ | r = .14 | >.6 | r = .27 | >.4 |
| /o:/ | r = .15 | >.6 | r = .17 | >.6 |
| /u:/ | r = .22 | >.4 | r = .18 | >.5 |
| Vowel F2 |  |  |  |  |
| /a/ | r = .50 | =.09 | r = .62 | =.03 |
| /a:/ | r = .31 | >.3 | r = .52 | =.08 |
| /e:/ | r = .59 | =.04 | r = .48 | >.1 |
| /ɛ/ | r = .70 | =.01 | r = .77 | =.01 |
| /ɪ/ | r = .63 | =.03 | r = .63 | =.03 |
| /i/ | r = .19 | >.5 | r = .46 | >.1 |
| /i:/ | r = .42 | >.1 | r = .28 | >.3 |
| /o/ | ρ = -.04 | >.8 | r = .07 | >.8 |
| /o:/ | r = .37 | >.2 | r = .32 | >.3 |
| /u:/ | r = .17 | >.6 | r = .30 | >.3 |
| Vowel duration |  |  |  |  |
| /a/ | r = .42 | >.1 | r = -.11 | >.7 |
| /a:/ | r = .57 | =.06 | r = .26 | >.4 |
| /e:/ | ρ = .72 | =.01 | ρ= .54 | =.06 |
| /ɛ/ | r = .38 | >.2 | r = -.12 | >.7 |
| /ɪ/ | r = ..46 | >.1 | r = .29 | >.3 |
| /i/ | r = .18 | >.5 | r = .03 | >.9 |
| /i:/ | r = .61 | =.04 | r = .44 | >.1 |
| /o/ | r = .14 | >.6 | r = .38 | >.2 |
| /o:/ | r = .14 | >.6 | r = -.20 | >.5 |
| /u:/ | r = .22 | >.4 | r = .57 | =.06 |
|  |  |  |  |  |
| /s/ measures |  |  |  |  |
| Center of gravity | ρ = .34 | >.2 | ρ = .37 | >.2 |
| skewness | r = -.26 | >.4 | r = -.27 | >.3 |
| kurtosis | ρ = -.27 | >.3 | r = -.26 | >.4 |
| duration | r = .59 | =.04 | r = .28 | >.3 |
|  |  |  |  |  |
| Speaking rate | r = -.25 | >.2 | r = .11 | >.7 |

Correlation between acoustic cues and speakers self-identification for the German sample.

|  | Correlation with Homo-index | *p* value |
| --- | --- | --- |
| Vowel F0 |  |  |
| /a/ | ρ = -.21 | >.5 |
| /a:/ | ρ = .10 | >.7 |
| /e:/ | ρ = -.41 | >.1 |
| /ɛ/ | ρ = -.33 | >.2 |
| /ɪ/ | ρ = -.27 | >.4 |
| /i/ | ρ = -.22 | >.4 |
| /i:/ | ρ = -.10 | >.7 |
| /o/ | ρ = -.30 | >.3 |
| /o:/ | ρ = -.41 | >.1 |
| /u:/ | ρ = -.18 | >.5 |
| Vowel F1 |  |  |
| /a/ | ρ = .66 | =.02 |
| /a:/ | ρ = .75 | =.01 |
| /e:/ | ρ = .07 | >.8 |
| /ɛ/ | ρ = .80 | <.001 |
| /ɪ/ | ρ = .39 | >.2 |
| /i/ | ρ = .33 | >.2 |
| /i:/ | ρ = .15 | >.6 |
| /o/ | ρ = .21 | >.5 |
| /o:/ | ρ = .17 | >.6 |
| /u:/ | ρ = -.06 | >.8 |
| Vowel F2 |  |  |
| /a/ | ρ = .21 | >.5 |
| /a:/ | ρ = .24 | >.4 |
| /e:/ | ρ = -.01 | >.9 |
| /ɛ/ | ρ = .30 | >.3 |
| /ɪ/ | ρ = .35 | >.2 |
| /i/ | ρ = -.01 | >.9 |
| /i:/ | ρ = .17 | >.5 |
| /o/ | ρ = -.18 | >.5 |
| /o:/ | ρ = -.11 | >.7 |
| /u:/ | ρ = -.07 | >.8 |
| Vowel duration |  |  |
| /a/ | ρ = -.06 | >.8 |
| /a:/ | ρ = .23 | >.4 |
| /e:/ | ρ = .24 | >.4 |
| /ɛ/ | ρ = .06 | >.8 |
| /ɪ/ | ρ = -.20 | >.5 |
| /i/ | ρ = .07 | >.8 |
| /i:/ | ρ = -.16 | >.6 |
| /o/ | ρ = -.37 | >.2 |
| /o:/ | ρ = .03 | >.9 |
| /u:/ | ρ = -.21 | >.5 |
|  |  |  |
| /s/ measures |  |  |
| Center of gravity | ρ = .42 | >.1 |
| skewness | ρ = -.46 | >.1 |
| kurtosis | ρ = -.40 | >.2 |
| duration | ρ = .03 | >.9 |
|  |  |  |
| Speaking rate | ρ = -.06 | >.8 |
