# Supplementary material for: The Sound of Voice: Voice-Based Categorization of Speakers’ Sexual Orientation within and across Languages
Source: PLoS One. 2015 Jul 1;10(7):e0128882. doi: 10.1371/journal.pone.0128882 (PMC4488841; doi:10.1371/journal.pone.0128882)
Supplement: S5 Table — Legend:. .1; * < .05; ** < .01; *** < .001; ns: not significant; Cluster: 1 = heterosexual; 2 = homosexual (DOC) [file pone.0128882.s005.doc]

Experiment 3A (Italian participants – Italian and German speakers). Mean ratings, analyses and cluster values for each speaker.

| Speaker | Self-reported SO | Language | Mean rating value  (standar deviation) | t-test | p value | Cluster |
| --- | --- | --- | --- | --- | --- | --- |
| Speaker 01 | heterosexual | Italian | 2.43 (1.03) | -9.19 | *** | 1 |
| Speaker 02 | heterosexual | Italian | 2.20 (0.92) | -12.46 | *** | 1 |
| Speaker 03 | heterosexual | Italian | 2.46 (1.14) | -8.03 | *** | 1 |
| Speaker 04 | heterosexual | Italian | 4.28 (1.28) | 5.36 | *** | 2 |
| Speaker 05 | heterosexual | Italian | 3.37 (1.32) | < 1 | ns | 1 |
| Speaker 06 | heterosexual | Italian | 3.93 (1.38) | 2.81 | ** | 2 |
| Speaker 07 | heterosexual | Italian | 2.31 (1.19) | -8.81 | *** | 1 |
| Speaker 08 | heterosexual | Italian | 3.86 (1.27) | 2.50 | * | 2 |
| Speaker 09 | heterosexual | Italian | 2.22 (0.84) | -13.35 | *** | 1 |
| Speaker 10 | heterosexual | Italian | 2.58 (0.98) | -8.30 | *** | 1 |
| Speaker 002 | heterosexual | German | 2.53 (1.13) | -7.61 | *** | 1 |
| Speaker 003 | heterosexual | German | 3.22 (1.16) | -2.07 | * | 1 |
| Speaker 007 | heterosexual | German | 3.77 (1.36) | 1.76 | . | 2 |
| Speaker 009 | heterosexual | German | 2.69 (1.23) | -5.79 | *** | 1 |
| Speaker 011 | heterosexual | German | 2.45 (1.12) | -8.21 | *** | 1 |
| Speaker 013 | heterosexual | German | 2.96 (1.25) | -3.79 | *** | 1 |
| Speaker 11 | homosexual | Italian | 2.75 (1.16) | -5.63 | *** | 1 |
| Speaker 12 | homosexual | Italian | 3.92 (1.30) | 2.88 | ** | 2 |
| Speaker 13 | homosexual | Italian | 2.91 (1.07) | -4.86 | *** | 1 |
| Speaker 14 | homosexual | Italian | 2.69 (1.12) | -6.35 | *** | 1 |
| Speaker 15 | homosexual | Italian | 2.86 (1.27) | -4.44 | *** | 1 |
| Speaker 16 | homosexual | Italian | 5.56 (0.71) | 25.89 | *** | 2 |
| Speaker 17 | homosexual | Italian | 5.40 (0.68) | 24.57 | *** | 2 |
| Speaker 18 | homosexual | Italian | 4.32 (1.18) | 6.22 | *** | 2 |
| Speaker 19 | homosexual | Italian | 3.36 (1.44) | <1 | ns | 1 |
| Speaker 20 | homosexual | Italian | 3.49 (1.26) | <1 | ns | 1 |
| Speaker 006 | homosexual | German | 4.96 (1.11) | 11.66 | *** | 2 |
| Speaker 015 | homosexual | German | 2.25 (1.09) | -10.15 | *** | 1 |
| Speaker 027 | homosexual | German | 2.45 (1.09) | -8.47 | *** | 1 |
| Speaker 029 | homosexual | German | 4.01 (1.14) | 3.96 | *** | 2 |
| Speaker 030 | homosexual | German | 3.86 (1.10) | 3.09 | ** | 2 |
| Speaker 031 | homosexual | German | 3.11 (1.20) | -2.83 | ** | 1 |

. < .1; * < .05; ** < .01; *** < .001; ns: non significant; cluster: 1 = heterosexual; 2 = homosexual

Experiment 3B (German participants – Italian and German speakers). Mean ratings, analyses and cluster values for each speaker.

| Speaker | Self-reported SO | Language | Mean rating value  (standar deviation) | t-test | p value | Cluster |
| --- | --- | --- | --- | --- | --- | --- |
| Speaker 01 | heterosexual | Italian | 3.21 (1.31) | -1.14 | ns | 2 |
| Speaker 02 | heterosexual | Italian | 2.50 (1.17) | -4.52 | *** | 1 |
| Speaker 03 | heterosexual | Italian | 3.21 (1.31) | -1.14 | ns | 2 |
| Speaker 04 | heterosexual | Italian | 4.07 (1.41) | 2.14 | * | 2 |
| Speaker 05 | heterosexual | Italian | 3.35 (1.41) | <1 | ns | 2 |
| Speaker 06 | heterosexual | Italian | 2.89 (1.39) | -2.30 | * | 1 |
| Speaker 07 | heterosexual | Italian | 2.60 (1.22) | -3.84 | ** | 1 |
| Speaker 08 | heterosexual | Italian | 3.64 (1.33) | <1 | ns | 2 |
| Speaker 09 | heterosexual | Italian | 2.42 (0.79) | -7.17 | *** | 1 |
| Speaker 10 | heterosexual | Italian | 2.57 (1.31) | -3.73 | ** | 1 |
| Speaker 002 | heterosexual | German | 2.85 (1.29) | -2.62 | * | 2 |
| Speaker 003 | heterosexual | German | 3.17 (1.02) | -1.66 | ns | 2 |
| Speaker 007 | heterosexual | German | 2.85 (1.11) | -3.05 | ** | 2 |
| Speaker 009 | heterosexual | German | 2.60 (1.19) | -3.94 | ** | 2 |
| Speaker 011 | heterosexual | German | 1.82 (0.86) | -10.29 | *** | 1 |
| Speaker 013 | heterosexual | German | 2.28 (0.85) | -7.52 | *** | 1 |
| Speaker 11 | homosexual | Italian | 1.96 (1.03) | -7.84 | *** | 1 |
| Speaker 12 | homosexual | Italian | 3.03 (1.40) | -1.71 | . | 1 |
| Speaker 13 | homosexual | Italian | 3.42 (1.31) | < 1 | ns | 2 |
| Speaker 14 | homosexual | Italian | 2.74 (1.22) | -3.21 | ** | 1 |
| Speaker 15 | homosexual | Italian | 2.32 (0.98) | -5.34 | *** | 1 |
| Speaker 16 | homosexual | Italian | 4.28 (1.38) | 3.00 | ** | 2 |
| Speaker 17 | homosexual | Italian | 3.71 (1.35) | < 1 | ns | 2 |
| Speaker 18 | homosexual | Italian | 3.39 (1.03) | < 1 | ns | 2 |
| Speaker 19 | homosexual | Italian | 2.60 (1.19) | -3.94 | ** | 1 |
| Speaker 20 | homosexual | Italian | 2.78 (1.19) | -3.15 | ** | 1 |
| Speaker 006 | homosexual | German | 3.75 (1.35) | < 1 | ns | 2 |
| Speaker 015 | homosexual | German | 2.25 (0.88) | -7.45 | *** | 1 |
| Speaker 027 | homosexual | German | 1.67 (0.66) | -14.39 | *** | 1 |
| Speaker 029 | homosexual | German | 3.10 (1.13) | -1.83 | . | 2 |
| Speaker 030 | homosexual | German | 3.07 (1.15) | -1.96 | . | 2 |
| Speaker 031 | homosexual | German | 3.10 (1.25) | -1.65 | ns | 2 |

. < .1; * < .05; ** < .01; *** < .001; ns: non significant; cluster: 1 = heterosexual; 2 = homosexual
